# Arteriolar degeneration and stiffness in cerebral amyloid angiopathy are linked to Aβ deposition and lysyl oxidase

**DOI:** 10.1002/alz.70254

**Published:** 2025-06-04

**Authors:** Lissa Ventura‐Antunes, Alex Nackenoff, Wilber Romero‐Fernandez, Yongchao Wang, Allison M. Bosworth, Alex Prusky, Emmeline Wang, Cristian Carvajal‐Tapia, Alena Shostak, Hannah Harmsen, Bret Mobley, Jose Maldonado, Neely Womble, Elena Solopova, J. Caleb Snider, W. David Merryman, Ethan S. Lippmann, Matthew Schrag

**Affiliations:** ^1^ Department of Neurology Vanderbilt University Medical Center Nashville Tennessee USA; ^2^ Department of Biomedical Engineering Vanderbilt University Nashville Tennessee USA; ^3^ Department of Pathology Vanderbilt University Medical Center Nashville Tennessee USA; ^4^ Vanderbilt Neurovisualization Lab Vanderbilt University Nashville Tennessee USA; ^5^ Vanderbilt Brain Institute Vanderbilt University Nashville Tennessee USA; ^6^ Department of Chemical and Biomolecular Engineering Vanderbilt University Nashville Tennessee USA; ^7^ Vanderbilt Memory and Alzheimer's Center Vanderbilt University Medical Center Nashville Tennessee USA

**Keywords:** Alzheimer's disease, amyloid beta, cerebral amyloid angiopathy, lysyl oxidase, three‐dimensional microscopy, vascular smooth muscle

## Abstract

**INTRODUCTION:**

The morphological and molecular changes associated with the degeneration of arterioles in cerebral amyloid angiopathy (CAA) are incompletely understood.

**METHODS:**

*Post mortem* brains from 26 patients with CAA or neurological controls were analyzed using light‐sheet microscopy, and morphological features of microvascular degeneration were quantified using surface volume rendering. Vascular stiffness was analyzed using atomic force microscopy.

**RESULT:**

Vascular smooth muscle cells (VSMCs) volume was reduced by ≈ 55% in CAA. This loss of VSMC volume correlated with increased arteriolar diameter, variability in diameter, and the volume of amyloid beta (Aβ) deposition in the vessel. Vessels with CAA were > 300% stiffer than controls. The volume of extracellular matrix cross‐linking enzyme lysyl oxidase (LOX) correlated closely with vascular degenerative features.

**DISCUSSION:**

Our findings provide valuable insights into the connections among LOX, Aβ deposition, and vascular stiffness in CAA. Restoration of physiologic extracellular matrix properties in penetrating arteries may yield a novel therapeutic strategy for CAA.

**Highlights:**

- We conducted 3D microscopy on human brains with cerebral amyloid angiopathy.
- We quantified features of vascular degeneration, β‐amyloid, and lysyl oxidase in CAA
- Vascular degeneration correlated with Aβ, loss of VSMCs , and increased LOX.
- Arterioles with CAA were stiffer than controls in data from atomic force microscopy.
- Vascular extracellular matrix properties may be a therapeutic target for CAA.

## 1 BACKGROUND

While neuritic plaques and neurofibrillary tangles are the hallmark lesions of Alzheimer's disease (AD), vascular pathology has been recognized as an important contributor to AD for > 100 years.^1^, ^2^ In the 1930s, amyloid material was found in cerebral and meningeal vessels. Two decades later, it was linked to AD and termed congophilic angiopathy due to its detectability with the Congo red stain.^3^, ^4^ Now known as cerebral amyloid angiopathy (CAA), this entity is defined by deposits of aggregated amyloid beta (Aβ) in cerebral blood vessels. Arterioles with CAA have disrupted architecture and a propensity toward hemorrhage, making CAA the second most common cause of intracerebral hemorrhage in the elderly after hypertension.^5^, ^6^, ^7^ CAA is more common with advancing age and frequently co‐occurs with AD; > 80% of patients with AD have some degree of CAA.^5^, ^8^ CAA is linked to cognitive impairment independent of the association with AD.^9^ Several autosomal dominant variants of CAA are known, but most cases are sporadic. Greater clinical awareness of CAA began in the 1990s as magnetic resonance imaging (MRI) techniques were developed that could detect small areas of bleeding in the brain termed cerebral microbleeds (CMBs). CMBs usually occur in a lobar distribution in CAA and are associated with white matter hyperintensities and enlarged perivascular spaces. This pattern has become the key diagnostic imaging feature of the disease.^10^ The recently updated Boston criteria provide excellent sensitivity and specificity for clinically diagnosing CAA.^11^

Morphologically, Aβ forms deposits primarily in the tunica media layer of arterioles, and as vessels with CAA degenerate, they lose vascular smooth muscle cells (VSMCs).^12^, ^13^ This process mechanically impairs vascular reactivity, leading to suboptimal autoregulation of blood flow in the brain,^14^ which has been seen in studies using arterial spin labeling (ASL) in patients with CAA and mouse models.^13^, ^15^ Prior reports suggested late complement activation and various extracellular matrix remodeling enzymes, including matrix metalloproteinases and lysyl oxidase (LOX), may play a role in vascular degeneration in CAA.^16^, ^17^, ^18^ Despite these molecular hints, surprisingly little is known about the mechanisms leading to vascular degeneration in CAA. Even the notion that Aβ plays a role in the progression of vascular degeneration has been questioned. Several authors independently reported that Aβ is not consistently present at sites of vascular rupture in CAA, creating doubts whether Aβ is a driving factor.^19^, ^20^

Because small vessel disease can contribute to cognitive decline and may be an important variable in patient selection for emerging therapies for AD and related dementias,^21^, ^22^ it is important to understand the mechanisms of vascular injury in CAA and identify therapeutic targets that address both the parenchymal and vascular features of AD.

We aimed to study the changes in vessel structure related to vascular degeneration and assess the correlation between Aβ deposition, microvascular degeneration, and CMB in CAA pathology. We also interrogated the biophysical properties of vessels with Aβ deposits and assessed the degree to which the extracellular matrix crosslinking enzyme LOX is associated with vascular degeneration. Defining the cellular and molecular features of microvascular degeneration in CAA is important to understanding vascular dysfunction's contribution to cognitive decline in AD and CAA.

## 2 METHODS

### 2.1 Study design

Brain tissue for this analysis was obtained from clinical autopsies performed at Vanderbilt University Medical Center and the University of California, Los Angeles (UCLA) Brain Bank, including neurological controls and patients with CAA over a range of severities. For each subject, we selected tissue samples from each cortical lobe (frontal, temporal, parietal, and occipital cortex) and obtained additional samples from any areas with visible CMBs. When large hemorrhages or strokes were present, those areas were excluded from tissue acquisition. All patients with CAA met clinical as well as autopsy criteria for CAA. Most cases were diagnosed via the Boston criteria with multiple lobar CMBs,^11^, ^23^ while a few were identified by computed tomography criteria after suffering multiple lobar hemorrhages.^24^ CAA severity was evaluated in each autopsy using the Vonsattel criteria with grades 0, 1, and 2 corresponding to absent CAA, mild, and moderate CAA, respectively, and grades 3 and 4 representing severe CAA.^25^ Cases were graded based on the pathologist's impression of the overall severity across cortical tissue blocks; CAA pathology can be patchy, so focal areas of higher severity were often observed within the studied cases, and this is noted in Table S1 in supporting information.

### 2.2 Optical tissue clearing

**FIGURE 1 alz70254-fig-0001:**
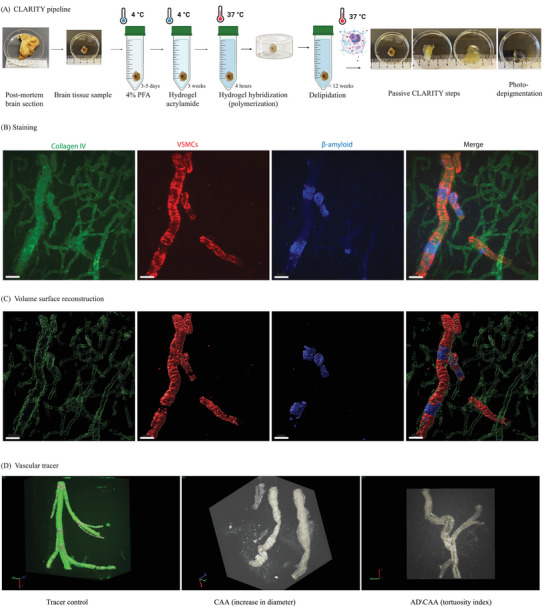
CLARITY methodology. A, Tissue handling pipeline for CLARITY applied to human tissue samples, *n* = 26 cases. B, Immunostaining of gray matter cortex from AD with moderate CAA, staining for vessels with Collagen IV‐488 in green, VSMCs in red, and methoxy‐X04 for Aβ in blue. Scale bar with 50 µm. C, Volume surface reconstruction from each of the images shown above using IMARIS software. Rotating three‐dimensional versions of the images in B–C are also viewable in Video S1 in supporting information. D, We manually traced each penetrating arteriole using Vesselucida 360 on the smart manual mode. The first panel shows the tracing of a control vessel, the second and third panels show examples with CAA. Aβ, amyloid beta; AD, Alzheimer's disease; CAA, cerebral amyloid angiopathy; VSMC, vascular smooth muscle cell.

The CLARITY methodology transforms opaque biological tissues into a nearly transparent hydrogel–tissue hybrid with preserved anatomical structure, proteins, and nucleic acids. The tissue becomes transparent after crosslinking to an infused hydrogel, providing a support framework for the brain tissue and lipid removal.^26^ We selected brain tissue samples of 1 to 3 cm^3^ thickness for tissue clearing (Figure 1A). Most tissue blocks were obtained at the time of death and were incubated for 3 to 5 days in 4% paraformaldehyde (PFA), larger specimens receiving a longer fixation time, and embedded in an acrylamide hydrogel (4% acrylamide, 0.05% bis‐acrylamide, 0.25% temperature‐triggering initiator VA‐044 in 0.1 M phosphate‐buffered saline in water–phosphate‐buffered saline [PBS]). Additionally, this study also used tissue samples that had been previously fixed as part of the clinical autopsy material and stored in 4% PFA for up to 3 months; these specimens received no additional fixation prior to embedding in acrylamide hydrogel. Adequate tissue incorporation of the hydrogel requires an oxygen‐free environment to prevent premature cross‐linking of acrylamide. To accomplish this, we placed the tissue sample in a 10 mL centrifuge tube and added enough hydrogel to fill the tube, leveraging surface tension so the hydrogel protruded above the lip of the tube when it was sealed with wax with no trapped air bubbles. The tissue blocks remained in the hydrogel at 4°C for 3 weeks, then were moved to a 37°C water bath for 4 hours to activate the VA‐044 acrylamide crosslinker and polymerize the hydrogel. After polymerization, the lipid component of the tissue was passively cleared with prolonged washing in a solution consisting of 0.2 M boric acid and 4% w/v sodium dodecyl sulfate (SDS) at pH 8.5 at 37°C with gentle agitation. The process of tissue clearing involved exchanging the buffer once or twice a week until the optimal translucence was achieved; typically between 8 and 12 weeks, but for specimens with longer fixation time, larger samples, and specimens with more blood in the parenchyma, the process could take longer. Upon completion, we transferred the tissue into PBS with 0.1% Triton‐X in a light booth equipped with a 1200 W LED array (BESTVA DC Series) for 24 to 48 hours of exposure to intense visible spectrum light at 4°C to photo‐depigment the tissue and suppress autofluorescence. The tissue was then washed and incubated in PBS with sodium azide 0.02% until staining. In Figure S1 in supporting information, we show representative examples of cleared specimens from neurologic controls (Figure S1A), mild/moderate CAA (Figure S1B), and severe CAA (Figure S1C) before immunostaining, which shows the variability of vessels in the same tissue block and the visibility of hemorrhage products at the severe CAA case. Additionally, in Figure S1D, we include an acquisition after the photo‐depigmentation process described above, using a lightsheet microscope, from the same severe CAA case shown in Figure S1C, showing minimal residual autofluorescence.

**RESEARCH IN CONTEXT**

1. **Systematic review**: Cerebral amyloid angiopathy (CAA) is defined by the accumulation of amyloid beta (Aβ) in the vessel wall and is associated with vascular fragility, but there is considerable doubt about the mechanism(s) of vascular fragility in CAA. Several studies have questioned whether Aβ is directly related to vascular degeneration.
2. **Interpretation**: Our study provides a systematic analysis of degenerative microvascular morphologies using novel three‐dimensional microscopy of human brain tissue. We found that microvascular degeneration was strongly correlated with Aβ and lysyl oxidase in cerebral vessels. Degenerating vessels exhibited markedly increased stiffness.
3. **Future directions**: Defining the molecular mechanisms of microvascular degeneration in CAA is essential for identifying new pharmacologic targets. LOX may be a future target in the treatment of CAA.

### 2.3 Immunostaining

#### 2.3.1 Staining of cleared tissue blocks

**FIGURE 2 alz70254-fig-0002:**
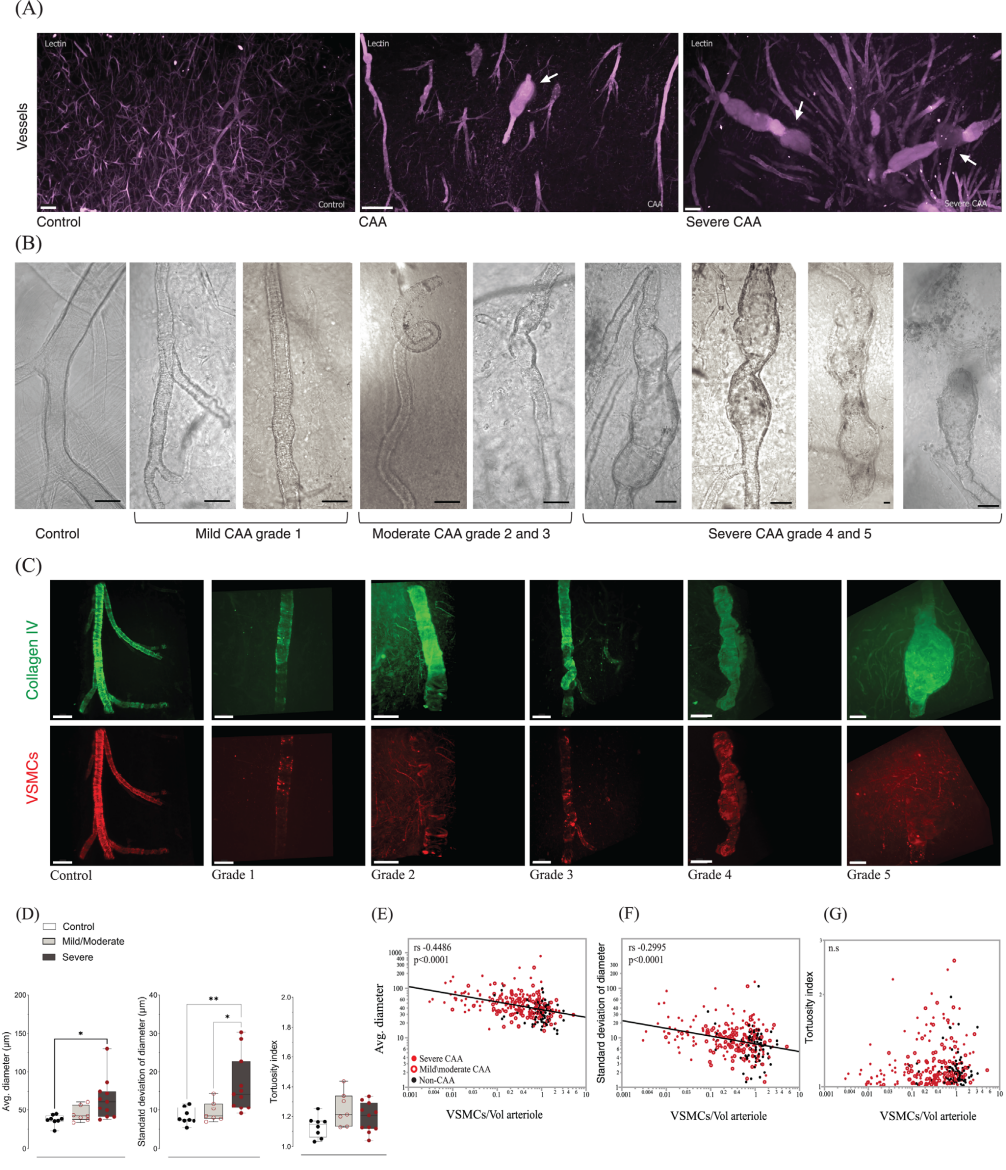
Morphological changes and grades of arterioles affected by CAA. A, Macro view of the cerebral microvascular network stained with tomato‐lectin, in purple. Left panel, cerebral vessels in a neurological control from gray matter cortex with intact vascular wall morphology. Central and right panels, vessels from subjects with CAA show degenerative vascular morphology with enlarged vessel diameter, areas of distention and in the third image CMBs (at arrows), both from gray matter cortex. Video S2 in supporting information shows three‐dimensional videos expanding on these three views. B, Phase‐contract imaging showing a range of levels of vascular degeneration in penetrating arterioles with CAA. C, Fluorescent images showing the relationship between morphological degeneration (stained with collagen IV‐488) and loss of VSMCs, in red. The control arteriole has a smooth vascular wall with intact VSMCs; mild, grade 1 degeneration is structurally intact with a thickened vessel wall; moderate degeneration, grades 2 or 3, has increased tortuosity and more pronounce VSMC loss, and severe degeneration, grades 4 or 5, has progressively worsening dilation, areas of aneurysm or stricture and, at the terminal stage, vascular rupture, scale bar 100 µm. D, Average arteriolar diameter and average variability of the lumenal diameter for each case significantly increased with CAA severity (by autopsy criteria) while the tortuosity of penetrating arterioles per cases was not significantly different across CAA severities (one‐way ANOVA, *P > 0.05. E, The Spearman correlation between average diameter, variability of diameter (SD), and tortuosity with VSMCs. Vascular dilation and the variability in the vascular diameter strongly correlate with VSMC loss, tortuosity peaks at an early stage of vascular degeneration. The average of VSMCs per case are described on Tables S3 and S4 in supporting information. ANOVA, analysis of variance; CAA, cerebral amyloid angiopathy; CMB, cerebral microbleed; SD, standard deviation; VSMC, vascular smooth muscle cell.

Optically cleared tissue blocks were washed overnight in PBS with 0.1% Triton‐X and preincubated with PBS containing 0.1% Triton‐X and 0.2 M boric acid at 37°C. To stain the blood vessels, we used a directly conjugated primary antibody against collagen IV mouse anti‐human, Alexa Fluor 488 (5 µg/mL, Cat# 53987182, Invitrogen), and incubated for 3 days in PBS with 0.1% Triton‐X. The tissue blocks were then washed and stained with conjugated antibodies for LOX (5 µg/mL, Cat# NB100‐2527AF647 Lox antibody Alexa fluor 647, Novus Biologicals, LLC). The specificity of the LOX antibody was validated as shown in Figure S2 in supporting information. We also stained with a mouse monoclonal antibody against VSMCs with α‐Smooth Muscle actin—Cy3 (5 µg/mL, Cat# C6198, Sigma). Amyloid was stained with methoxy‐X04, incubated overnight (1:500 from in 5 mM in distilled H2O, Cat # 4920, Tocris Bioscience), as indicated in the figure legends and Video S1 in supporting information. The samples were then washed in PBST 0.1% at 37°C. To validate the staining pattern obtained with methoxy‐X04 was consistent with the presence of Aβ, we co‐stained two samples (one with CAA, one with AD without CAA) with a mouse monoclonal antibody to Aβ (1:200, Cat# MCA‐AB9, EnCor Biotechnology, Figure S3 in supporting information) which demonstrated reasonable concordance between the staining approaches. To visualize the vascular network on a larger scale, the three‐dimensional images in Video S2 in supporting information and the associated illustrative pictures in Figure 2A were stained using tomato‐lectin (5 µg/mL, Cat# FL1171, VECTOR) to stain vessels using the same protocol above.

#### 2.3.2 Tissue sectioning and staining for laser scanning microscopy

Free‐floating sections[Fig alz70254-fig-0001] of[Fig alz70254-fig-0002] *post mortem* human tissue were prepared on a Leica 1200 S vibratome with 50 µm or 100 µm thickness; 100 µm sections were used to visualize the culprit vessel within CMBs in tissue blocks, and the remainder of the analyses used 50 µm sections. Aβ was detected using thiazine red (1 µM, Cat# 2150‐33‐6, Chemsavers), nuclei with DAPI (0.5 µM, Cat# 1485, Vector lab), and a mouse monoclonal antibody against VSMCs α‐smooth muscle actin (α‐Smooth Muscle Actin, Cat# A5228), associated with corresponding secondary antibody, Alexa Fluor 488 (Thermo Fisher A21206). Confocal images were acquired through the Vanderbilt Cell Imaging Shared Resource (CISR) using a Zeiss LSM 710 confocal laser‐scanning microscope.

### 2.4 Light sheet fluorescence microscopy

All optically cleared tissue blocks used in the quantitative analysis were stained using the same protocol; methoxy‐XO4 to visualize Aβ, collagen immunostaining to visualize the vascular network, α‐smooth muscle actin–Cy3 (Cat# C6198, Sigma) and LOX (Cat# NB100‐2527AF647, Novus Biologicals, LLC). After immunostaining, optically cleared tissue samples were incubated overnight in 68% thiodiethanol (TDE; Cat# 166782, Sigma) with a refractive index of 1.33 to match the optical diffraction of the microscope. Three‐dimensional images were acquired with a Z1 lightsheet microscope (Carl Zeiss) at the Vanderbilt CISR with 405 nm, 568 nm, 488 nm, and 638 nm lasers, using 20× objective illumination (Objective Clr Plan‐Neofluor 20×/1.0 Corr nd = 1.45 M32 85 mm) and high‐resolution scientific cameras that allow exceptional image quality and precise measurements (PCO Edge sCMOS). We acquired images of vessels in the arteriolar size range (15–120 µm) and with anatomy reasonably conforming to the expectations of a penetrating arteriole (containing actin staining of the tunica media and/or amyloid and running from the surface of the brain toward the white matter). Within a block of tissue, we attempted to image all the relevant vessels for which good‐quality images could be obtained (vessels with clear damage from handling or small segments were omitted) to represent the full spectrum of pathological vessels. Three‐dimensional image stacks were assembled from plane‐by‐plane image collection in Z‐plane depth between 750 and 2050 µm using ZEN software (Zeiss) for processing. In addition, larger scale images of vascular networks obtained from optically cleared tissue specimens (Figure 2A and Video S2) were obtained on a SmartSPIM single‐plane illumination light sheet microscope (LifeCanvas Technologies) with a 3.6× objective (NA  =  0.2) and axially swept rolling shutter with 488 nm lasers. After acquisition, images were transferred to a Dell Precision 7920 Tower with (2) Intel Xeon Gold 5218R CPU at 2.1 GHz and 526 GB of RAM running Windows 10 Pro for Workstations for stitching and processing. Images were stitched into Z‐stacks with a modified version of Terastitcher Keyframe. Animations from the original image data were generated in Imaris (version 10, Bitplane, Oxford Instruments).

### 2.5 Image analysis process and quantification

Vascular images produced via light sheet fluorescence microscopy (LSFM) from the CLARITY tissue blocks were reconstructed, and surface volume was rendered and quantified using the software Imaris (as shown in Figure S1). We used automated detection of objects of interest to estimate features of vessel geometry (stained with collagen IV), including surface volume of VSMCs, average diameter, and variability in diameter. Vascular Aβ volume (stained with methoxy‐X04), vascular LOX volume were also estimated. Image parameters used to measure the surface volumes included adjustments to account for immunostaining signal intensity including: (1) smoothing details set at 0.688, (2) thresholding adjusted to optimally recognize each voxel of immunostaining, and (3) rendering each structure volume (vascular volume, VSMCs volume, vascular Aβ, and vascular LOX) using the absolute intensity with background subtraction. Image size was ≈ 30 GB with 16‐bit images; size: X:1920 µm Y:1920 µm Z:800 µm to 900 µm. The Z size varied according to the object of interest, and the volume of the sample voxel size was ≈ X 0.344 Y:0.344 Z: between 0.500 to 0.700. To trace vessels, we analyzed the three‐dimensional images acquired at the LSFM using the Vesselucida 360 software (MBF Bioscience) to determine vessel length, tortuosity index, and diameter. The arterioles were traced using the smart manual mode (Figure 1D). The Vesselucida 360 software was limited in its ability to trace severely damaged vessels, so those with rupture or significant discontinuity in staining were omitted from the assessment of tortuosity, and we estimated the length and volume of these arterioles with IMARIS using the vascular fragments to estimate their geometry. For intact vessels, the software Vesselucida Explorer (MBF Bioscience) was used to calculate the number of segments, the average diameter, length, and tortuosity index. The segment is modeled as a series of frusta, and the total length of the path is used to trace the segment. To estimate the average diameter, the software calculates the length‐weighted mean. To estimate the tortuosity index, the ratio of the actual length of the segment to the shortest distance between the endpoints of the segment is calculated so that a straight segment receives a value of 1, and as tortuosity increases and the segment assumes a more complex path to reach its destination, the value will increase, typically ranging as high as a value ≈ 2.5.

### 2.6 Atomic force microscopy

Atomic force microscopy (AFM) was performed on 30 µm thick *post mortem* unfixed human tissue sections. Prior to imaging, sections were incubated with 1 µg/mL Hoechst (Cat# H1399, ThermoFisher) and 2 µM thiazine red (Cat# 2150‐33‐6, Chemsavers) for 5 minutes at room temperature to label nuclei and amyloid aggregates, respectively. Sections were mounted on a Nikon Eclipse Ti microscope and submerged in PBS. Penetrating arterioles were identified through brightfield and fluorescence channels. The Bioscope Catalyst AFM was mounted onto the microscope platform to measure vessel stiffness. AFM probes (Bruker Nano MLCT‐bio‐drift compensated) with a spring constant of 0.03 N/m were calibrated, and cross‐sections through vessel walls were scanned at a frequency of 0.25 Hz in tapping mode, capturing scan areas of 4 to 10 µm within the vessel wall. A total of six vessels were measured from each case, selected from at least two different sections. Each scan consisted of 16,384 elastic modulus measurements, which were averaged, and the resulting six averages were plotted as a box and whisker plot.

### 2.7 Statistical analysis

The arteriolar surface volume, VSMCs, vascular Aβ, and LOX volumes were calculated from surface rendering using IMARIS. Once the object surface was created, the volumes were recorded using automated tools as shown in Figure 1C. Vascular diameter, length, and tortuosity were generated by Vesselucida Explore. Because CAA is a patchy pathology affecting some arterioles more than others, even when they are in close proximity, for most analyses, each penetrating arteriole was treated as an independent measurement.

Correlations between vascular volume, LOX, Aβ, and morphological features/tracing measurements were performed in JMP17 2.0 software. Non‐parametric Spearman correlation coefficients with a pairwise line fit were used to get a robust measure of Spearman correlation; for visual representation in the figures, we used a pairwise linear fit. *p* value < 0.05 was considered statistically significant. In addition, one‐way analysis of variance (ANOVA) was applied to the correlation between cases and the average of diameter, the SD of diameter, and tortuosity. The difference between CAA and non‐CAA vascular stiffness was compared using a Student *t* test in GraphPad Prism software version 10 (GRAPH PAD Software Inc.). We assessed the association of sex with vascular morphological features using one‐way ANOVA in Figure S3. In addition, given the hierarchical structure of our data and the variability specific to each case, we used mixed‐effect models to fit our data using the “lmer 4” function from the “LmerTest” package in R. The model can be expressed as:

$$Y_{ij}=\beta_{0}+\beta_{1}*X_{ij}+\beta_{2}*Sex+\beta_{3}*Age+\gamma_{i}+\in_{ij}$$

where *Y_ij_* represents the response variable for the *j*th observation in the *i*th group; β_0_​ is the intercept; β_1_ is the fixed effect coefficient for predictor X_ij_; β_2_​ and β_3_ are the fixed effect coefficients for sex and age, respectively; γ_i_​ represents the random effect capturing deviations specific to the *i*th group; and ϵ_ij_ denotes the error term for the *j*th observation in the *i*th group.^27^, ^28^

## 3 RESULTS

We analyzed 384 images from LSFM of 26 human *post mortem* brains, including 11 cases with severe CAA, 7 cases with mild/moderate CAA (5 with concurrent AD, 2 with frontotemporal dementia), and 8 cases that served as neurological controls, without CAA, AD, or frontotemporal dementia. We performed three‐dimensional microscopy on optically cleared tissue blocks and digitally created surface renderings of the images of cortical arterioles affected by different grades of CAA to evaluate morphological alterations in the vessels. This workflow is illustrated in Figure 1 and Video S1.

### 3.1 Microvascular degeneration

Because of the uncertainty about whether Aβ is causative of vascular degeneration in CAA, we aimed to characterize the features of vascular degeneration in CAA agnostic to the presence of Aβ. Cortical arterioles from neurological control cases had smooth, thin, uniform vessel wall appearance, but we observed a range of morphological features in optically cleared specimens from patients with CAA which we felt were likely evidence of vascular degeneration (see the large‐scale reconstruction of samples from a neurological control and two CAA cases shown in Figure 2A and Video S2). These features included vascular tortuosity, dilation of penetrating arterioles, and variability in the caliber of arterioles (with both strictures and microaneurysms) and are illustrated in Figure 2A, B. We aimed to quantify these features and assess their association with VSMC loss in the arteriolar wall, which is a well‐established degenerative feature of CAA.^29^ Tortuosity, lumenal diameter, and variability in the lumen size were all detectable with LSFM using collagen IV staining as a primary marker (Figure 2C). These features seemed to represent a spectrum of vascular degeneration which, for the purposes of our analyses, we defined in five grades ordered according to our perception of their severity. Healthy‐appearing arterioles were designated as grade 0. Arterioles with widening of the lumen but that remained straight with smooth contours were designated grade 1, then with mild dilation and increased tortuosity grade 2, then with increased variability in the vessel diameter and loss of the smooth vessel‐wall contour and tortuosity grade 3, then with lumenal dilation and variability in lumen size grade 4, and finally, with the emergence of marked structural degeneration including aneurysm and rupture grade 5 (Figure 2B, C). We found there was good agreement between raters using this scoring scale (average Kappa = 0.805). Arterioles from neurological controls had an average diameter of 36.40 ± 2.24 µm (mean ± standard error of the mean), while the average diameter was 48.65 ± 3.33 µm for mild/moderate CAA and 62.49 ± 4.32 µm in donors with severe CAA (one‐way ANOVA *F* = 5.522, *P* = 0.0110), and variability of vascular diameter similarly increased with CAA severity (one‐way ANOVA *F* = 7.01, *P* = 0.0042) demonstrating that assessment of morphological features of vascular degeneration roughly paralleled the clinical assessment of CAA severity, details per cases is demonstrated in Table S2 in supporting information.

### 3.2 Relationship of vascular smooth muscle to degenerative morphologies

To further assess the validity of this grading scheme for vascular degeneration and ensure it correctly ordered the features of arteriolar degeneration, we evaluated the correlation between morphological features of degeneration with VSMC loss. The loss of VSMCs from arterioles as CAA progresses is probably the most established and reliable marker of vascular degeneration in CAA at this time.^12^, ^30^ Three‐dimensional microscopy of control tissue showed arterioles had intact VSMCs covering a high percentage of the vessel wall, while the VSMC signal was reduced in vessels with CAA as the morphological alterations of the vascular wall progressed (Figure 2B, C and Video S1), as others have previously reported.^31^ The volume of VSMCs associated with the arteriole was reduced by 55% in cases with CAA compared to non‐CAA cases. We hypothesized that the average arteriolar diameter would increase with the loss of VSMCs, and we found a strong inverse correlation between these two variables *(r_s_* (279) = 0.4486,  *p*< 0.0001; Figure 2E). In Figure S4A in supporting information, we analyzed average arteriolar diameter and VSMCs separated by sex and found no difference. Additionally, we applied a mixed‐effect model to our analysis, incorporating both age and sex to adjust for their impact on the correlation between average arteriolar diameter and VSMCS. The results of the mixed‐effect model (β_1_ = −0.00237, *P* = 0.0088) showed that the correlation is robust to the effects of age and sex.

Areas of stricture and dilation cause irregularity in the lumen, which can be quantified as an increase in the variability in the average arteriolar diameter. We hypothesized that the variability in arteriolar diameter would increase with loss of VSMCS. Our analyses found that the variability in vessel diameter was inversely correlated with VSMC volume (*r_s_* (279) = −0.2995, *p*< 0.0001; Figure 2F). The variability in vessel diameter was not significantly different between sexes (Figure S4B). The correlation between the standard deviation of diameter and VSMCs was not statistically significant in a mixed‐effects model adjusted for age and sex (*β*_1_ = −0.00237, *P* = 0.0329). However, the variability in the diameter of penetrating arterioles was greater in patients with an autopsy diagnosis of severe CAA compared to neurological controls and mild/moderate CAA (Figure 2D).

Finally, there was no significant correlation between tortuosity and VSMC volume across the entire cohort (Figure 2G). In an exploratory analysis, we found there was a weak correlation of tortuosity with VSMCs when only cases with CAA were included, peaking at an early disease stage (*r_s_* (236) =  0.1787, *p* = 0.0096). Because tortuosity peaked at an early stage of VSMC loss, we interpreted it as an early feature of vascular degeneration.

### 3.3 Association of Aβ with microvascular degeneration

**FIGURE 3:**
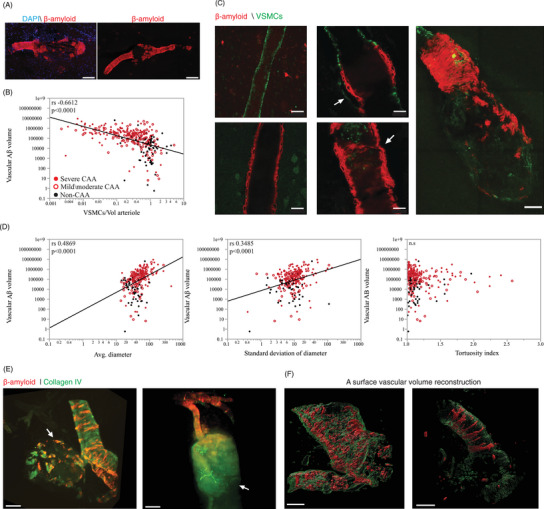
Association of Aβ accumulation in the wall of the arteriole and structural degeneration and rupture. A, Laser scanning microscopy of tissue sections stained for Aβ with thiazine red (red) and nuclei in DAPI (blue) from gray matter sections from CAA cases, 100 µm scale bar. B, Spearman correlation between vascular Aβ surface reconstruction and VSMCs, see also Tables S3 and S4 in supporting information. C, Staining of 50 µm sections through vessels with and without CAA shows the absence of staining of VSMCs in areas with heavy Aβ accumulation. The arrow in the upper middle image shows VSMCs outside the tunica media, consistent with a fibrotic process. The arrow in the lower middle image indicates the site of a focal stricture in the vessel wall, scale bar 100 µm. D, Spearman correlation between vascular Aβ and average diameter, variability of diameter, and tortuosity. E, F Three‐dimensional images of ruptured arterioles with staining of Aβ with thiazine red (red) and collagen IV‐488; the first two images show native fluorescence, the last two show a vascular surface rendering. Disorganized “shards” of Aβ are present near the site of each rupture, scale bar 100 µm. Detailed three‐dimensional views of these ruptured vessels can be seen in Video S4 in supporting information. Aβ, amyloid beta; CAA, cerebral amyloid angiopathy; VSMC, vascular smooth muscle cell.

Next, we assessed the degree to which vascular Aβ deposition correlated with morphological features of vascular degeneration (Figure 3A). We quantified vascular Aβ volume on penetrating arterioles in cortical specimens for comparison to the morphological features of degeneration identified in the first stage of this study. Vascular Aβ volume strongly correlated with the loss of VSMCs across the cohort (*r_s_* (297) =*= −*0.6612, *p* < 0.0001; Figure 3B). The volume of vascular Aβ was not significantly different when stratified by sex (Figure S4C). The mixed‐effect model adjusted for age and sex confirmed the observed correlation between vascular Aβ volume and VSMCs (*β*_1 _= −1.34e‐07, *P* = 0.0004). Spatial comparisons of the location of Aβ deposits and VSMC staining consistently showed that VSMCs were lost from vessel segments with heavy Aβ deposition (see Figure 1B, C, and Video S1). The same pattern was observable in 50 µm thick tissue sections imaged with laser scanning confocal microscopy (Figure  3C). In most affected vessels, Aβ formed a ring‐shaped structure encircling the vessel wall within the tunica media, and this ring, in many cases, had a central void that lacked amyloid staining. This central void was occasionally stained for VSMCs (Figure 3C), but in most cases, VSMC staining was absent. In areas with patchy Aβ deposits, we observed that the rings of Aβ were in line with VSMC staining. These observations led us to conclude that the Aβ rings probably form around VSMC cells, which subsequently involute, a hypothesis supported by previous studies.^32^ We also observed occasional VSMC staining in vessels with CAA outside of the tunica media, which we hypothesized might be evidence of splitting of the vessel wall or else vascular fibrosis (see arrows in Figure 3C), a topic which should receive additional research attention in the future.

Next, we assessed the relationship between vascular Aβ volume and vessel diameter (demonstrated in Video S3 in supporting information), and we found they were strongly correlated (*r_s_* (241) =  0.4869, *p* < 0.0001). Similarly, the variability in the diameter of vessels correlated with vascular Aβ volume (*r_s_* (243) =  0.3485, *p*< 0.0001; Figure 3D), suggesting that vessels lose their smooth barreled morphology as Aβ deposition progresses. This correlation between vascular Aβ volume and both vessel diameter and variability in vascular diameter remained significant in a mixed‐effect model adjusted for age and sex (*β*_1 _= 6809.7, *P* = 5e‐08 and *β*_1_ = 18,510.8, *P* = 3.3e‐9, respectively). Vascular Aβ volume did not correlate with tortuosity across the entire cohort, but in an exploratory analysis was modestly correlated with the index of tortuosity when vessels with CAA were evaluated alone (*r*_s_ (196) =  −0.1923, *p* = 0.0038), peaking at an early stage of Aβ deposition.

### 3.4 CMBs and Aβ

We next attempted to address whether vascular Aβ is associated with CMBs. We identified 78 CMBs in our specimens, which were visible on gross tissue inspection, either on natural or cut surfaces, or after tissue clearance (see Figure S1); the culprit vessel was easily detectable running through or adjacent to the blood products. CMB ranged in size from a few hundred microns in diameter to several millimeters and appeared black, brown, or dark red. We also examined 13 severely dilated vessels that seemed near to rupture. In morphologically intact vessels with Aβ deposition, Aβ formed ring‐shaped structures organized orthogonally to the direction of blood flow. In ruptured arterioles and those with severe focal dilations, the organized, ring‐shaped deposits were disrupted into sharply contoured “shards” of Aβ. Others have described that Aβ deposits in CAA are rigid,^14^ so we interpreted this “shard‐like” appearance as a shattering of the rings of Aβ. The expansion of the volume of these vascular segments could lead to the impression that Aβ deposition was proportionally decreased in traditional microscopy of thin tissue sections, but in every CMB we imaged, Aβ was present. Figure 3E, Video S3, and Video S4 in supporting information show ruptured arterioles and shards of Aβ deposits in vessels with CAA vessels. Figure 3F, along with the corresponding Video S4, shows an example of volume reconstruction of ruptured vessels and the presence of Aβ at these sites. The percentage of vascular Aβ volume and the percentage of VSMCs/arteriole volume are both presented in Table S3 in supporting information and the coefficient correlation in Table S4 in supporting information.[Fig alz70254-fig-0004]

### 3.5 Atomic force microscopy assessment of vessel stiffness in CAA

To assess whether cortical arterioles with Aβ deposition are stiffer than vessels without CAA, we performed AFM on *post mortem* brain tissue from a subset of our cases: five cases with CAA and five controls (Table S1). AFM enables observation of surfaces at the nanoscale by scanning with a tiny probe mounted on a cantilever, which is run over the surface of a sample, generating a surface profile with exceptional resolution.^33^, ^34^ In addition to producing ultra‐high‐resolution images, this technology allows for the examination of biomechanical properties and is able to measure tissue stiffness through nanomechanical force measurements.^35^

**FIGURE 4 alz70254-fig-0004:**
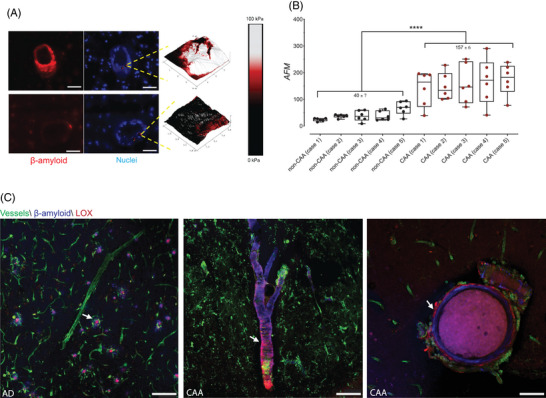
AFM and the stiffness of arterioles. A, Coronally sectioned profiles of arterioles from cases with CAA and without CAA were stained with thiazine red and DAPI. Cross‐sections of the vessel wall were evaluated by AFM to determine tissue stiffness (example density maps across the vessel wall are shown for each condition). Scale bar 50 µm. B, Average stiffness in kPa for non‐CAA (40.81 ± 7.19) and CAA cases (157.4 ± 6.25) is shown. C, Increased tissue stiffness could be partially mediated by the activity of extracellular matrix crosslinking enzymes like LOX. Confocal microscopy images of vessels stained in green, LOX (magenta), and Aβ staining with methoxy‐X04 (blue) show substantial LOX deposition in CAA, especially in degenerating arterioles (rightmost image). Aβ, amyloid beta; AFM, atomic force microscopy; CAA, cerebral amyloid angiopathy; LOX, lysyl oxidase.

To perform AFM, DAPI and thiazine red staining of frozen sections were used to identify vessels that had been cut cross‐sectionally during tissue sectioning, and stiffness measurements were made across the cut surface of the vessel wall. We found that vessels containing heavy Aβ deposits were significantly stiffer than vessels in control tissue, with mean stiffness values of CAA vessels ranging from 167 to 175 kPa while control vessels ranged from 36 to 68 kPa. Vessels with CAA were on the order of 4‐fold stiffer than vessels from donors without CAA (Figure 4A). The average AFM measurement per case in non‐CAA (40.81 ± 7.19 kPa) and CAA (157.4 ± 6.25 kPa) is shown in Figure 4B.

### 3.6 LOX and microvascular degeneration in CAA

The enzyme LOX is a driver of vascular fibrosis and cross‐links extracellular matrix polymer proteins to stiffen the connective tissue matrix of vessels.^36^ Its role in the brain is not well studied, although a few reports indicate it may be associated with Aβ pathology in AD and may be expressed in astrocytes.^37^, ^38^, ^39^ We hypothesized that LOX activity may contribute to the increased vessel stiffness observed in arterioles with CAA.

**FIGURE 5:**
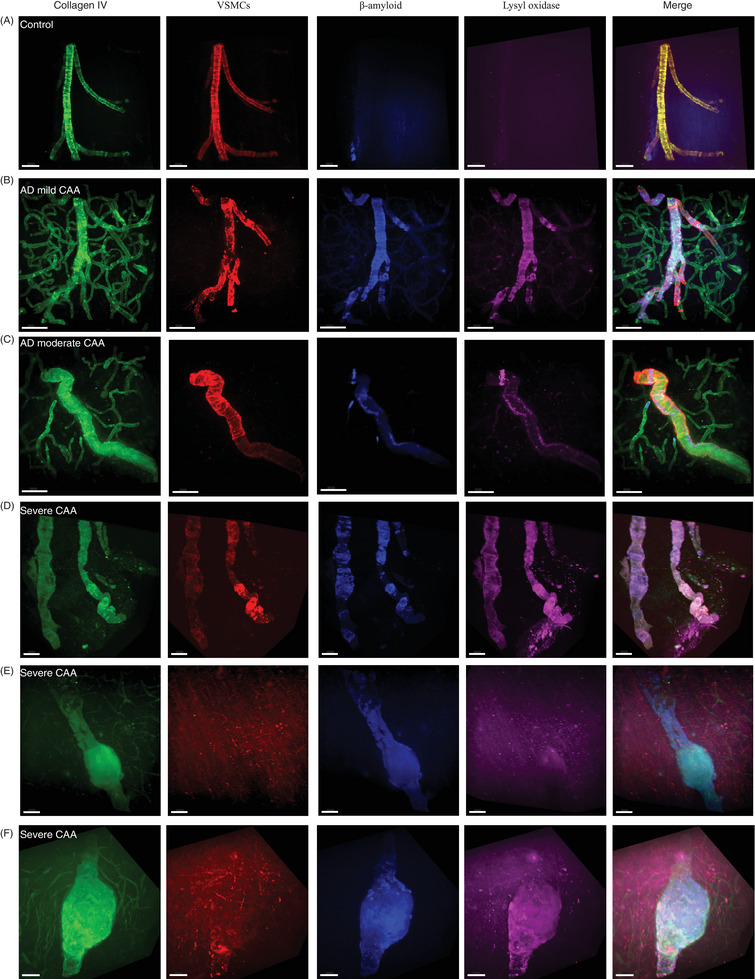
Morphological changes in vessels affected by CAA in relation to Aβ and LOX. Three‐dimensional images of a control arteriole are compared to mild, moderate, and severe CAA arterioles stained with collagen IV‐488 in green, VSMC actin‐ Cy3 in red, methoxy‐XO4 for Aβ, in blue and LOX‐ 647 in far‐red (shown in pink). A merged image of the four channels is shown in the last column, scale bar = 100 µm. A, Control with intact collagen IV and VSMCs, without methoxy‐X04 or LOX. B, Mild CAA shows a mild degeneration with patches of loss of VSMCs correlating with foci of Aβ and LOX staining. C, AD with moderate CAA shows areas of degeneration of VSMCs with deposition of Aβ and LOX correlating with twisting distortions of the arteriole. D, CAA with severe degeneration shown near‐complete loss of VSMCs with heavy deposition of Aβ and LOX, tortuosity and dilation of the arteriole. E and F, In severely degenerated arterioles with CAA, the deposits of Aβ lose their organized ring shape and look fractured at sites with aneurysmal dilation of the arteriole. Some autofluorescence is present in vessels that have ruptured or are severely dilated, likely due to residual blood products. The on‐target staining is brighter than the autofluorescence and the autofluorescence is present across a range of wavelengths. Aβ, amyloid beta; AD, Alzheimer's disease; CAA, cerebral amyloid angiopathy; LOX, lysyl oxidase; VSMC, vascular smooth muscle cell.

**FIGURE 6:**
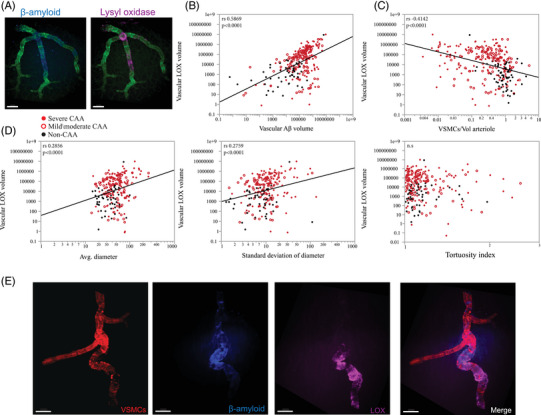
Morphological alteration of arteriolar walls correlates with volume of LOX. A, Aβ in blue stained with methoxy‐X04 and LOX in pink, showing the overlap of Aβ and LOX on the arteriole, scale bar with 100 µm. B, The volume of LOX strongly correlated with vascular Aβ (C) and inversely correlated with VSMC volume. D, LOX also correlated with increased vascular diameter and variability of the standard deviation of the diameter, while the total volume of LOX did not correlate with tortuosity. E, Eccentric deposits of LOX were frequently observed at sites of severe tortuosity. Aβ is stained in blue stained with methoxy‐X04; VSMC actin in red; LOX in pink, scale bar 100 µm. See also Video S5 and Tables S3 and S4 in supporting information. Aβ, amyloid beta; LOX, lysyl oxidase; VSMC, vascular smooth muscle cell.

We directly conjugated an anti‐LOX antibody for efficient staining of the tissue blocks and thoroughly validated its specificity for immunostaining (Figure S2). We discovered that LOX was present in some Aβ plaques, as had previously been reported.^40^ LOX was also present in vessels with CAA, primarily in the adventitia and especially in dilated, degenerating arterioles, as shown in the section in Figure 4C. We observed that LOX was often not directly colocalized with Aβ but was nevertheless spatially related to Aβ deposits, and we aimed to quantify this relationship. We stained optically cleared tissue blocks for collagen IV, methoxy‐X04 (Aβ), VSMCs, and LOX as in the previous section. Representative imaging results across grades of vascular degeneration are shown in Figure 5. We quantified the relationship between the LOX staining and morphological features of degeneration, as shown in Figures 2 and 3 and Tables S3 and S4. We assessed vascular LOX levels by sex and found no difference (Figure S4D). Vascular LOX was strongly correlated with Aβ abundance in the vasculature, with Spearman correlation (*r_s_* (268)  = 0.5869, *p* < 0.0001; Figure 6A‐B). Vascular LOX was inversely related to the presence of VSMCs (*r*_s_ (276)  = −0.4142, *p* < 0.0001; Figure 6C). LOX also correlated with vascular diameter (*r_s_* (231)  = 0.2854, *p* < 0.0001), and the variability in vascular diameter (*r_s_* (231)  = 0.2759, *p* < 0.0001; Figure 6D). In a mixed‐effect model adjusted for age and sex, vascular LOX remained robustly correlated with vascular Aβ volume (*β*_1 _= 0.846, *p* < 2e‐16). Vascular LOX also correlated with VSMC volume in this mixed‐effects model (*β*_1 _= −8.55e‐8, *p* = 0.037). Vascular LOX also remained significantly correlated with both average vessel diameter and variability in vessel diameter in a mixed effects model with adjustment for sex and age (*β*_1 _= 4146.2, *p* = 0.00056 and *β*_1 _= 15,011.9, *P* = 8.2e‐07, respectively).

Vascular tortuosity peaked with lower levels of LOX, but no statistically significant correlation was seen across the cohort. However, we found focal, asymmetric areas of LOX staining in arterioles were often present in twisted and distorted arteriole segments, and we hypothesized that asymmetric stiffening of the extracellular matrix may account for some of the arteriolar tortuosity observed in mild/moderate CAA, while heavier/circumferential areas of LOX reactivity have a symmetric effect on the vessel architecture (see Figure 6E and Video S5 in supporting information).

## 4 DISCUSSION

We conducted quantitative morphological analyses of vascular degeneration in CAA, confirming a strong association of loss of VSMCs with vascular degeneration and Aβ deposition in arterioles. We also discovered that vessels with CAA were markedly stiffer than vessels without CAA, which may be partially due to the effects of LOX on the extracellular matrix (Video S5). At early stages of degeneration, vessels were often tortuous, which we attributed to the presence of focal, eccentric deposits of Aβ and LOX, while at later stages, when both Aβ and LOX deposits tended to be extensive and circumferential, the vessels were generally straight. Strictures and aneurysmal dilations marked degenerating arterioles, and vascular Aβ deposits were consistently present at sites of vessel wall rupture. Vessels associated with CMB were focally dilated near the site of hemorrhage, with depletion of VSMCs and fractured shards of Aβ in the vessel wall (Video S4). A strength of this study is the use of quantitative three‐dimensional microscopy.

Neuropathology has traditionally relied on thinly cut slices of brain tissue to enable light penetration through the tissue for microscopy. Advances in confocal and multiphoton imaging enabled better light penetration and the ability to study thicker sections, but still, significant tissue processing remains, which can introduce artifacts and complicate the interpretation of microscopic findings. Historically, three‐dimensional tissue structure has been inferred by analyzing sequential tissue sections, sometimes with digital reconstruction, but this approach is cumbersome, and significant artifacts usually remain. This limitation is particularly problematic when studying networks (neuronal pathways or vascular networks) on a microscopic scale. The introduction of optical clearing techniques^26^ permitted the study of relatively larger blocks of tissue rendered transparent for superior light penetration. Optical tissue clearance for three‐dimensional microscopy is regularly performed upon mouse brain tissue and other experimental model systems^41^, ^42^, ^43^ but is rarely performed upon aged human brain tissue, and the few successful reports have mostly been on small sections. We developed strategies to manage the high level of autofluorescence from accumulated pigments in brain tissue to ambitiously apply this innovative technique to study the cerebral microvascular network in the aged human brain.

Compared to neuronal tau tangles and parenchymal plaques, the contribution of vascular pathologies to AD has been relatively understudied. Most patients with AD have some degree of CAA in autopsy studies, and 40% have some form of vascular cognitive impairment and dementia (VCID).^44^, ^45^, ^46^, ^47^ Behind AD, VCID is the second most common cause of dementia. CMBs detected on MRI in a lobar distribution are a well‐established clinical biomarker for CAA. CMBs are linked to the Aβ burden on positron emission tomography amyloid imaging and cerebrospinal fluid markers of β‐amyloidosis, and they predict both hemorrhage risk and deterioration of cognitive function.^48^ For this reason, the association of CMBs with CAA is not in doubt. However, the mechanism of hemorrhage in CAA is incompletely understood. The most prevalent assumption is that Aβ is directly toxic to various elements of the arteriole, leading to fragility and hemorrhage, and there is significant in vitro and in vivo evidence to support this view.^49^ However, previous reports that Aβ was absent at vascular rupture sites could question this hypothesis. An alternative hypothesis could be proposed that loss of VSMCs due to Aβ deposition in more proximal arterioles could lead to hyperperfusion from loss of autoregulation and turbulent flow due to disruption of the lumenal architecture, leading to vascular remodeling, including distention and hemorrhage.

Several studies have questioned the association between vascular β‐amyloidosis and CMBs. In one cohort study, three recent and four old CMBs were identified, with Aβ present in only one.^18^ The authors interpreted hemorrhage products in the perivascular space as a CMB. We previously reported that hemorrhage from a CMB could propagate in the perivascular space some distance from the CMB, suggesting the evaluated tissue did not include the vessel segment that ruptured.^50^ In another case, CMB in the white matter was examined,^19^ but Aβ deposition in CAA is largely restricted to the cortical ribbon. It is not surprising that the host vessel did not demonstrate Aβ deposition.^51^ Moreover, they interpreted microscopic perivascular hemosiderin deposits as CMB, but these are too small to produce the CMB detected by MRI and probably represent a distinct phenomenon. Finally, in a third study, no Aβ was present around CMBs in the putamen, but CAA is not thought to be a typical cause of CMBs in deep nuclei;^52^ this is usually the sequelae of severe hypertension. In our study, we included subjects with severe CAA and numerous CMBs and examined the tissue with three‐dimensional imaging of optically cleared tissue, which enabled confident identification of CMBs and the culprit vessel. Our results support the hypothesis that Aβ may directly compromise vessel integrity, though other mechanisms may also be involved.

Morphologically intact vessels with CAA had organized rings of Aβ around VSMC fibers. When vessels become significantly dilated or rupture, these rings appear to shatter, leaving behind sharply contoured fragments or shards of Aβ. Because of the aneurysmal expansion in the arteriolar volume at rupture sites, the amount of Aβ in proportion to the vessel size may be fairly small, but it is consistently present. What role the loss of the Aβ ring structure plays in the latter stages of vascular degeneration remains unproven, but it is a consistent feature of severely degenerated arterioles in CAA.

The enzyme LOX is responsible for matrix remodeling and stiffening in blood vessels.^53^ LOX is necessary for blood vessel development, as shown in morphogenesis studies in a mouse model.^54^ Atherosclerotic lesions contain pathological levels of LOX, and loss of LOX is associated with intracranial aneurysm rupture.^55^, ^56^, ^57^ Administration of irreversible pan‐LOX inhibitor aminopropionitrile significantly diminished the stiffness and tensile strength of aortic tissue in rats.^58^ We discovered that the volume of LOX on cerebral vessels strongly correlated with degenerative morphologies and Aβ deposition. Cross‐linking of the extracellular matrix mediated by pathologic levels of LOX likely accounts in part for the increased vascular stiffness we observed in CAA. LOX is also a well‐known driver of vascular fibrosis, which could contribute to increased vascular stiffness. LOX catalyzes the oxidation of susceptible lysine residues, thereby producing highly reactive aldehyde groups that spontaneously form covalent bonds with oxidized and unoxidized lysine residues nearby, leading to cross‐linking of LOX substrates, such as collagen and elastin fibrils, which ensures structural stability of the extracellular matrix.^59^ In the brain, LOX is expressed primarily by astrocytes, VSMCs, and endothelial cells.^60^ In addition to its role in vascular biology, LOX is implicated in cell adhesion^61^ and migration mechanisms in human disease, notably in hypoxia‐induced tumor metastasis.^62^, ^63^ LOX activity was reported to be increased in *post mortem* brains of patients with AD and dementia compared to control subjects.^40^ Furthermore, LOX protein levels were increased in *post mortem* AD brains, as analyzed in a systematic review.^64^ In AD, LOX was spatially associated with Aβ plaques and vessels with Aβ in a rare hereditary form of CAA.^18^

### 4.1 Limitations of the study

In neuropathological studies, it is difficult to establish causal relationships between the studied factors. Consequently, our interpretation of the role of Aβ and LOX in vascular degeneration is informed by existing findings in the literature and will need to be confirmed in mechanistic studies. Because the tissue used was sourced from clinical autopsies, there is some heterogeneity in the tissue handling. Most specimens were collected at the time of death, and tissue fixation was limited to 3 to 5 days. Some specimens, however, were obtained after a period of fixation not exceeding 3 months to permit completion of the autopsy evaluation. None of the tissue in this study had been in long‐term storage in a fixative, but we cannot exclude the possibility that variability in fixation time introduced some variability in the analysis. The main concern from overfixation is loss of antigenicity and increased autofluorescence. The staining protocol did not appear to be affected by fixation, and we introduced a photodepigmentation step to control autofluorescence. Nevertheless, some autofluorescence was still present, particularly around areas of hemorrhage. This autofluorescence was fainter than the signal from the staining probes and was present across multiple channels, making it easy to distinguish from true staining. We were able to threshold out most of the non‐specific signal, but we cannot exclude the possibility that autofluorescence could have modestly influenced the quantitative measures in specimens with hemorrhage products. Additionally, tissue sampling focused on capturing CMBs, so the anatomical regions included from each case are heterogeneous, but we feel this was well justified to ensure inclusion of severe pathology, which was likely to inform the features of microvascular degeneration.

### 4.2 Conclusion

This study shows a close relationship between the severity of Aβ deposition features of vascular degeneration, including VSMC depletion and hemorrhage. Moreover, this study extends our understanding of the role of LOX in neurodegeneration by convincingly showing that arteriolar degeneration in CAA and vascular Aβ volumes are linked to the extent of LOX deposition in arterioles. The role of LOX in vascular degeneration is worthy of further study.

## CONFLICT OF INTEREST STATEMENT

The authors L.V.A., A.N., W.R.F., Y.H., A.M.B., A.P., E.W., C.C.T., A.S., H.H., B.M., J.M., N.W., E.S., J.C.S., and W.D.M. declare no conflicts of interest. M.S.S. is funded by the NIH (R03NS111486‐01, R01AG078803, RF1NS130334, RF1NS129735, and R56AG07429) and has received consulting fees from Labaton Suscharow LLP, Raymond James Inc, Greycourt, and Guidepoint Inc. E.S.L. is funding by NIH research support (RF1NS129735, RF1NS130334, RO1AG092015, R01NS110665, R21NS137563, R21AG077807), Chan Zuckerberg Initiative, Ben Barres Early Career Acceleration Award (grant 2019‐191850), NSF grant 2033800. Author disclosures are available in the supporting information.

## CONSENT STATEMENT

Brain tissue donation was approved by the institutional review board (IRB) of Vanderbilt University Medical Center, and written informed consent was obtained from patients or their surrogate decision makers. All specimens used in the study were de‐identified. The study received IRB oversight as part of our ongoing Observational Study of Cerebral Amyloid Angiopathy and Related Disorders (OSCAAR, IRB# 180287). Deidentified tissue specimens sourced from the UCLA brain bank received similar institutional ethical oversight and were graciously provided by Dr. Harry V. Vinters. The study has been carried out in accordance with the Code of Ethics of the World Medical Association (Declaration of Helsinki).

## Supporting information



Supporting information

Supporting information

Supporting information

Supporting information

Supporting information

Supporting information

Supporting information

Supporting information

Supporting information

Supporting information

Supporting information

Supporting information

Supporting information

Supporting information

Supporting information
