# Supplementary material for: Arteriolar degeneration and stiffness in cerebral amyloid angiopathy are linked to Aβ deposition and lysyl oxidase
Source: Alzheimers Dement. 2025 Jun 4;21(6):e70254. doi: 10.1002/alz.70254 (PMC12136096; doi:10.1002/alz.70254)
Supplement: Supplementary file 2 — Supporting information [file ALZ-21-e70254-s014.docx]

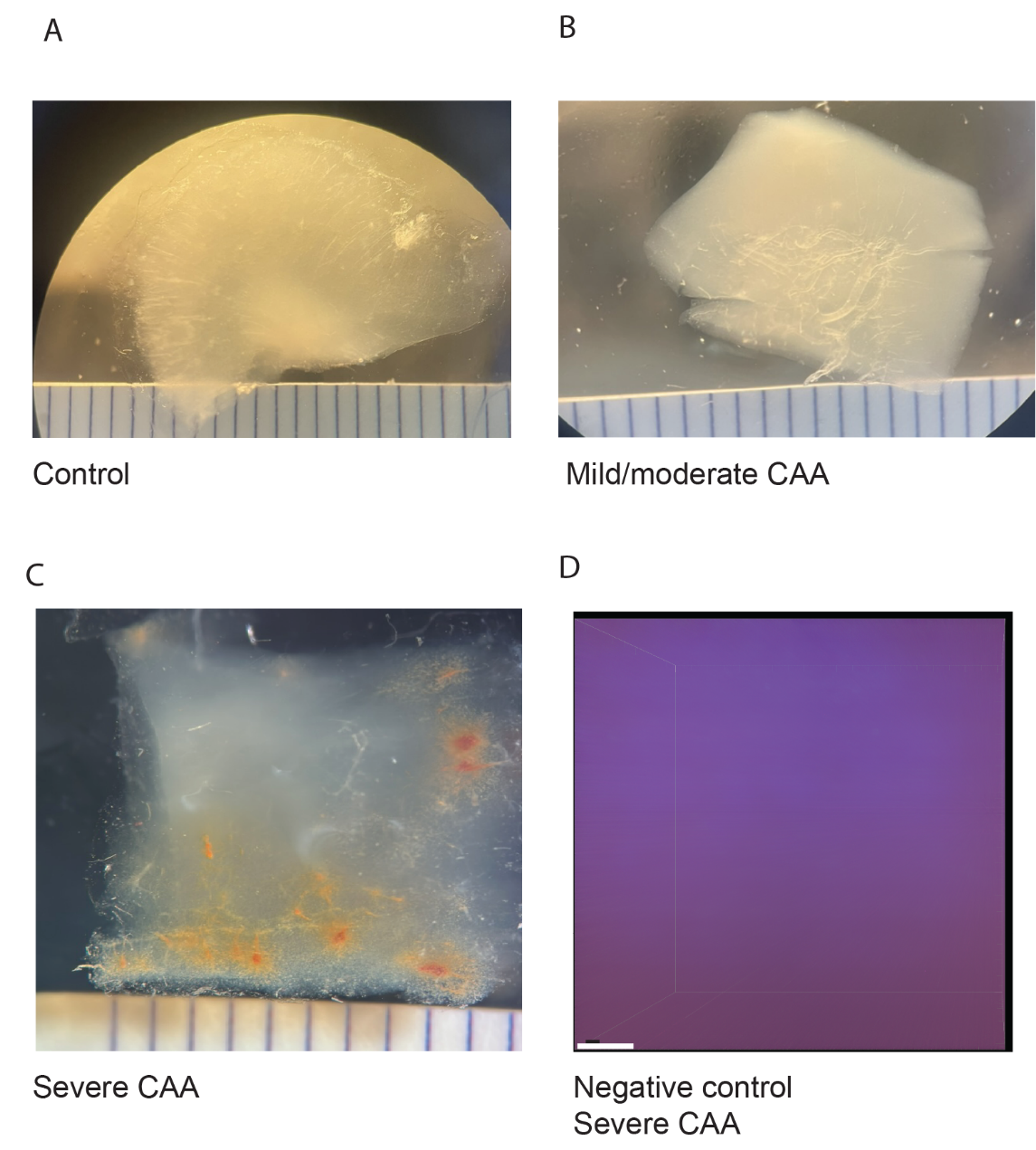


**Supplementary figure 1:** Tissue clearing and visualization of CMBs. **(A)** Control tissue post-clearing showing the transparent block of tissue with health vessels. **(B)** Brain tissue with mild/moderate CAA post-clearing showing tortuosity of vessels and **(C)** severe CAA post-clearing showing numerous hemorrhages surrounding abnormal appearing vessels. **(D)** Negative control (from the step after photo-depigmentation light treatment and before immunostaining) from a case with severe CAA, scale bar 100 µm. This is from the same case as shown in supplementary figure 1C, and figure 5E and 5F.
