# Supplementary material for: Arteriolar degeneration and stiffness in cerebral amyloid angiopathy are linked to Aβ deposition and lysyl oxidase
Source: Alzheimers Dement. 2025 Jun 4;21(6):e70254. doi: 10.1002/alz.70254 (PMC12136096; doi:10.1002/alz.70254)
Supplement: Supplementary file 3 — Supporting information [file ALZ-21-e70254-s002.docx]

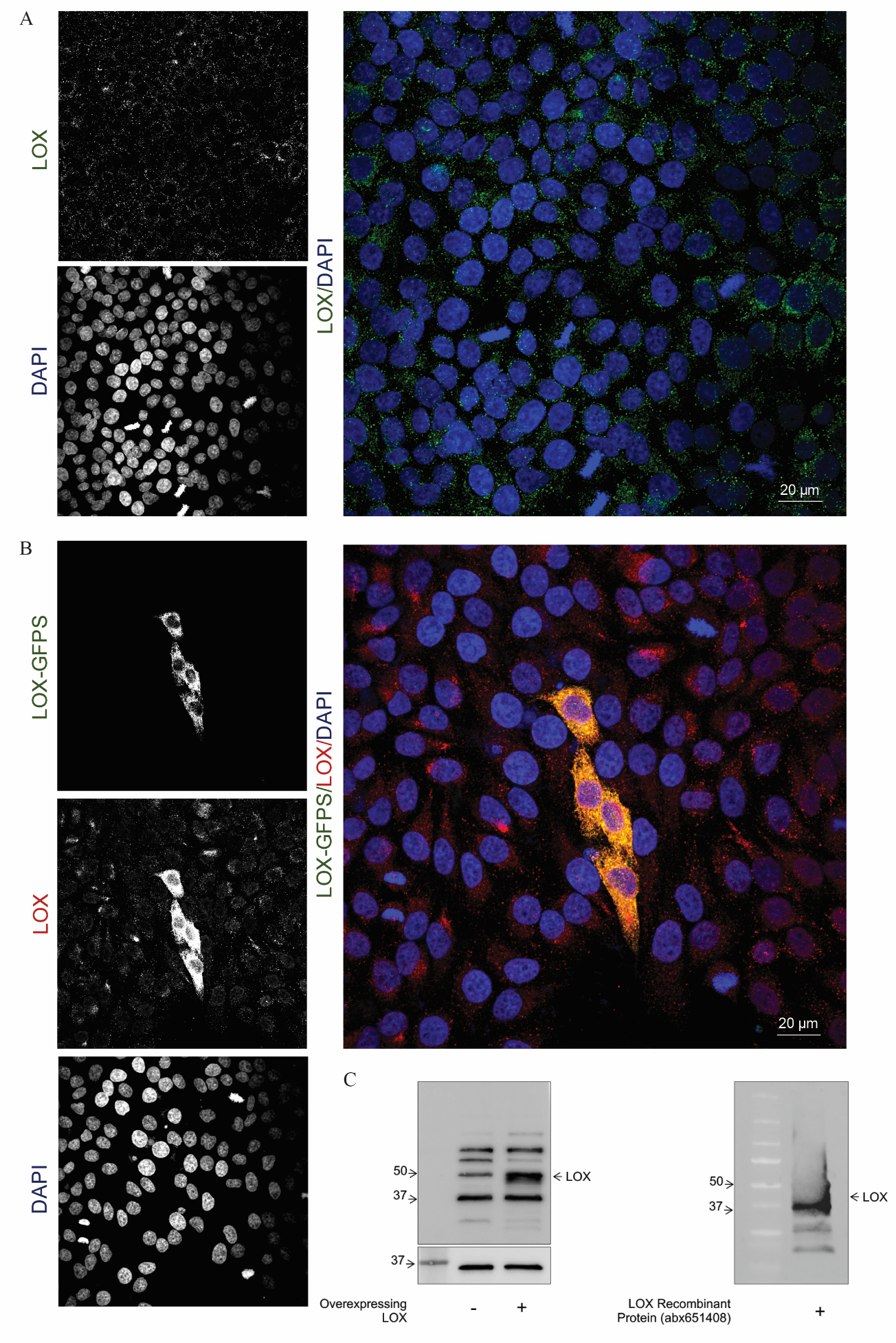


**Supplementary Figure 2:** Immunocytochemistry validation of the rabbit anti-LOX antibody **(A)** Image shows endogenous LOX levels (green). **(B)** Counterstained image shows immunodetection of LOX (red) in cells that were transfected with pCMV-LOX-GFPS (green). Yellow/orange fluorescence indicates areas where the antibody recognized the fluorescently labelled LOX. Images are from 5 μm z-stack scanning with a step interval of 1 μm, n=3. Nuclei were stained with DAPI. **(C)** Antibody validation by western blot analysis. *Left panel:*LOX was transient expressed in HeLa cells and 20 ug of cell lysates were resolved by SDS-PAGE and western blot was performed as described in methodology section. The increase in the intensity of the band corresponding to the expected molecular weight for LOX suggests that the antibody was able to recognize the recombinantly expressed LOX protein. *Right panel:* the anti-LOX antibody recognized mainly a single band corresponding to a purified recombinant LOX obtained from Abbexa (651408) (right panel).
