# Supplementary material for: Arteriolar degeneration and stiffness in cerebral amyloid angiopathy are linked to Aβ deposition and lysyl oxidase
Source: Alzheimers Dement. 2025 Jun 4;21(6):e70254. doi: 10.1002/alz.70254 (PMC12136096; doi:10.1002/alz.70254)
Supplement: Supplementary file 4 — Supporting information [file ALZ-21-e70254-s010.docx]

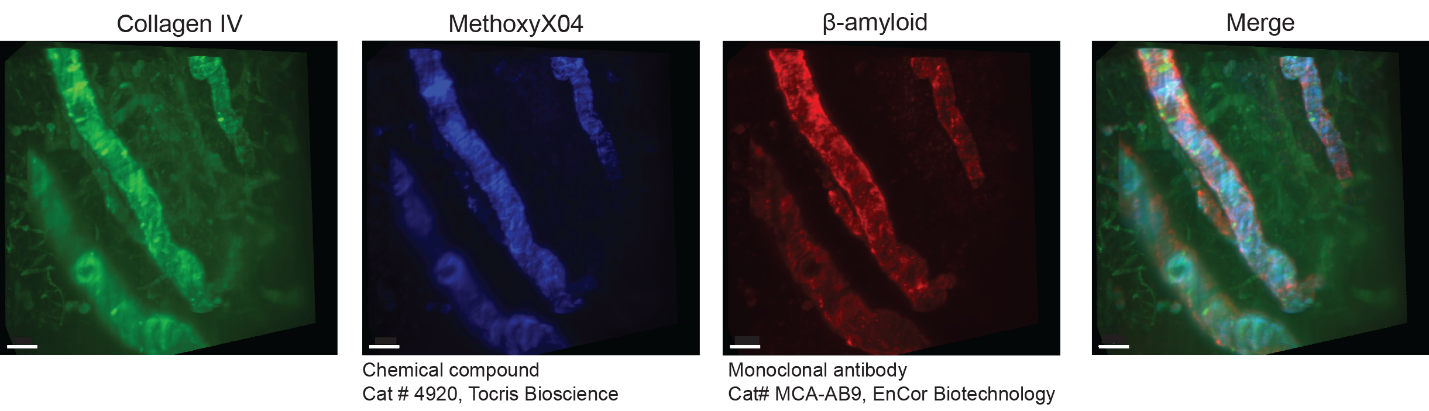


**Supplementary figure 3:** Comparison between the detection of β-amyloid by a monoclonal antibody (Cat# MCA-AB9, EnCor Biotechnology) versus methoxy-X04 (Cat # 4920, Tocris Bioscience). In blue (methoxy-X04), red (monoclonal β-amyloid) green (collagen IV) and a merged image, scale bar 50 µm. Methoxy-XO4 binds aggregated β-amyloid whereas the antibody will bind aggregates, soluble β-amyloid and amyloid precursor protein. The incomplete concordance between these stains is expected, but they are sufficiently concordant to support the use of methoxy-XO4 in this study.
