# Supplementary material for: Arteriolar degeneration and stiffness in cerebral amyloid angiopathy are linked to Aβ deposition and lysyl oxidase
Source: Alzheimers Dement. 2025 Jun 4;21(6):e70254. doi: 10.1002/alz.70254 (PMC12136096; doi:10.1002/alz.70254)
Supplement: Supplementary file 5 — Supporting information [file ALZ-21-e70254-s006.docx]

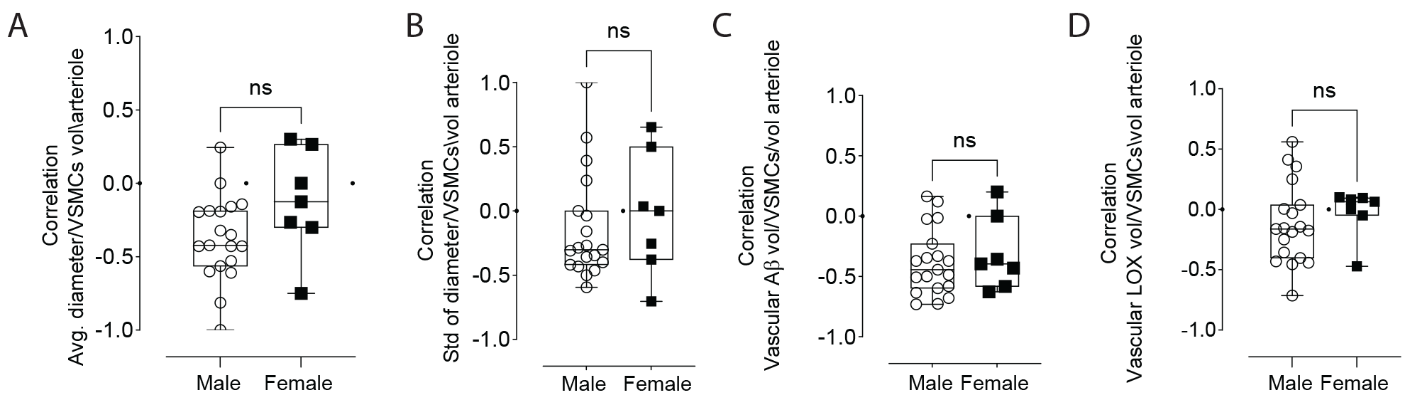


**Supplementary Figure 4:** CAA-associated vascular changes do not vary by sex in this cohort or between correlations of average diameter with VSMCs **(A)**; variability of diameter (std) with VSMCs **(B)**; vascular Aβ with VSMCs **(C)**; and vascular LOX with VSMCs **(D)**.
