# Supplementary material for: Arteriolar degeneration and stiffness in cerebral amyloid angiopathy are linked to Aβ deposition and lysyl oxidase
Source: Alzheimers Dement. 2025 Jun 4;21(6):e70254. doi: 10.1002/alz.70254 (PMC12136096; doi:10.1002/alz.70254)
Supplement: Supplementary file 6 — Supporting information [file ALZ-21-e70254-s007.docx]

**Supplementary methodology**

**Supplementary methodology figure 2:**

Immunocytochemistry validation of LOX antibody (Novus; NB100-2527) in HeLa cells. Cells were fixed, permeabilized and stained for LOX followed by Donkey Anti-Rabbit IgG H&L Alexa Fluor®488 or Alexa Fluor®594 and DAPI.  Hela cells were transfected with 2 ug of LOX plasmid pCMV3-LOX (HG17796-UT, Sino Biological) using the guidelines FuGENE HD protocol, then the cell proteins were extracted and 20 ug of cell lysates were denatured, separated, and transferred via western Blot following the protocol previously described. To check LOX protein expression, we used Rabbit anti-LOX antibody (1 ug/ml) (NB100-2527, NOVUS) and Mouse anti-GAPDH antibody (1 ug/ml) (ab59164, Abcam). The images were obtained using secondary fluorescent antibodies (1:2500) (LICOR) in the Chemidoc MP Imaging System (BIO-RAD). Next, 5 ug of LOX recombinant protein (abx651408, Abbexa) were denatured, separated, and transferred using the Western blot protocol previously described. To check LOX protein expression we used Rabbit anti-LOX antibody (0.5 ug/ml) (NB100-2527, NOVUS). The images were obtained using Goat anti-Rabbit-HRP secondary antibody (100 ng/ml) (65-6120, Invitrogen) and SuperSignal West Femto Maximum Sensitivity Substrate in the Chemidoc MP Imaging System (BIO-RAD). *Methods:* HeLa cells (American Type Culture Collection, United States) were grown in Dulbecco’s modified Eagle’s medium supplemented with 2 mM l-glutamine, 100 units/ml penicillin/streptomycin, and 10% (v/v) fetal bovine serum (FBS) at 37°C in an atmosphere of 5% CO2.  Cells were plated in 6-well plates onto coverslip at a concentration of 1x106 cells/well and cultured overnight prior to transfection. Cells were transiently transfected with 1 µg of pCMV-human LOX-GFPS (HG17796-ACG; Sino Biological) using FuGENE® Transfection Reagent according to the manufacturer's instructions.  For the immunocytochemistry cells with/without transfection were fixed in 4% paraformaldehyde, washed with TBS containing 20 mM glycine, permeabilized with 0.1% Triton X and labelling LOX with NB100-2527 (2 µg/mL) at 4°C overnight. After cells were washed and stained with Donkey Anti-Rabbit IgG H&L Alexa Fluor®488 or Alexa Fluor®594 at 1:1000 for 1h at room temperature. Nuclei were stained with DAPI and after extensively washed cells were mounted in a Vectashield immunofluorescence medium (Vector Laboratories, United States). Microscope observations were performed with a 63x oil immersion objective using a Zeiss LSM 710 Confocal Microscope (Zeiss, United States).
