# Supplementary material for: Arteriolar degeneration and stiffness in cerebral amyloid angiopathy are linked to Aβ deposition and lysyl oxidase
Source: Alzheimers Dement. 2025 Jun 4;21(6):e70254. doi: 10.1002/alz.70254 (PMC12136096; doi:10.1002/alz.70254)
Supplement: Supplementary file 7 — Supporting information [file ALZ-21-e70254-s013.docx]

**Supplementary table 1**

**Cases information and pathology description.**

| *Category* | *Case ID* | *Age/*  *Sex* | *Neuropathology* | *Cause of death* | *PMI* |
| --- | --- | --- | --- | --- | --- |
| *Severe CAA* | CAA 1 | 90+M | Severe CAA, focal cerebellar CAA. A1B1C0. Rare microinfarcts. | Bronchopneumonia / sepsis | 48h |
| *Severe CAA* | CAA 2 / AFM case 6 | 62M | Intracerebral hemorrhage and severe CAA. APOE 3/4, A0B1C0. | Right frontal and left parietal hemorrhage | 35h |
| *Severe CAA* | CAA 3 | 86M | Intracerebral hemorrhage, severe CAA, severe AD – A3B3C3. | Intracerebral hemorrhage and pulmonary embolus | 18h |
| *Severe CAA* | CAA 4 / AFM case 8 | 71M | Intracerebral hemorrhage, severe CAA, severe AD – A3B3C3, remote right parietal infarct. | Right frontal hemorrhage | 12h |
| *Severe CAA* | CAA 5 / AFM case 9 | 81M | 3cm right frontal hemorrhage, severe CAA, AD pathology A2B1C2. | Urinary tract infection in hospice care for end-stage dementia | 19h |
| *Severe CAA* | CAA 6 / AFM case 7 | 68F | Severe CAA including cerebellum, severe AD – A3B3C3. | Hospice care for end-stage dementia | 9h |
| *Severe CAA* | CAA 7 | 61M | Severe CAA, AD pathology A2B3C3, numerous microinfarctions, subarachnoid siderosis | Intracerebral hemorrhage | 10h |
| *Severe CAA* | CAA 8 | 69M | Severe CAA, no Alzheimer’s pathology. Scattered infarcts. | Sepsis, heart disease | 18h |
| *Severe CAA* | CAA 9 | 75F | Severe CAA, AD pathology A3B1C1. | GI infection (in hospice care). Remote episode of inflammatory CAA | 12h |
| *Severe CAA* | CAA 10 | 75M | Severe CAA, AD pathology A3B1C1. Subarachnoid siderosis, microinfarctions | Intracerebral hemorrhage | 38h |
| *Severe CAA* | CAA 11 | 81F | Severe CAA, Braak stage VI plaque pathology | End stage dementia | 21h |
| *Severe CAA* | AFM case 10 | 65F | Severe CAA, Braak stage VI plaque pathology | End stage dementia | 19h |
| *Mild/mod CAA* | CAA/AD 1 | 81F | Moderate CAA, severe AD pathology A1B3C3. Scattered cerebral infarctions | Heart disease and severe dementia | 36h |
| *Mild/mod CAA* | CAA/AD 2 | 68F | Mild/moderate CAA, severe AD – A3B3C3 | In hospice care for end-stage dementia | 15h |
| *Mild/mod CAA* | CAA/AD 3 | 67F | Moderate (focally severe) CAA, Braak stage VI plaque pathology | End-stage dementia | 28h |
| *Mild/mod CAA* | CAA/AD 4 | 66M | Moderate CAA, severe AD – A3B3C3. Brainstem predominant Lewy body disease | End-stage dementia in hospice care | 50h |
| *Mild/mod CAA* | CAA/mixed dementia 5 | 82M | Mild/moderate CAA. AD pathology – A3B0C2. LATE (TDP-43 stage 3/3). Diffuse Lewy body disease, hippocampal sclerosis | End-stage dementia in hospice care. | 8h |
| *Mild/mod CAA* | CAA/FTD 6 | 66M | Moderate CAA. Frontotemporal lobar degeneration due to TDP43 (AD pathology scoring A3B0C0). | Behavioral variant frontotemporal dementia | 10h |
| *Mild/mod CAA* | CAA/FTD 7 | 71M | Mild/moderate CAA. Frontotemporal lobar degeneration due to TDP43 (AD pathology scoring A3B1C0). | Corticobasal degeneration in hospice care | 6h |
| *No CAA* | Control 1 | 62M | Craniotomy site from remote subdural hemorrhage, microinfarction. | Portal vein thrombosis, sepsis | 11h |
| *No CAA* | Control 2 | 45M | Intracerebral atherosclerosis | Basilar artery stroke | 13h |
| *No CAA* | Control 3 | 87M | Left frontoparietal lacunar infarct, scattered rare neuritic plaques. | Myocardial infarction | 12h |
| *No CAA* | Control 4 | 87M | Left MCA stroke, Alzheimer’s pathology scoring A1B1C0. | Stroke (contralateral hemisphere used in study) | 6h |
| *No CAA* | Control 5 / AFM case 5 | 66F | Vertebrobasilar atherosclerosis with pontine and cerebellar infarcts. | Pulmonary emboli, stroke. | 33h |
| *No CAA* | Control 6 | 72M | Left cerebellar lacunar infarction | Acute respiratory distress syndrome, heart failure. | 12h |
| *No CAA* | Control 7 | 68M | None (Alzheimer’s pathology scoring A1B0C0) | Cardiogenic shock from intraoperative bleeding. | 24h |
| *No CAA* | Control 8 | 66M | None | Pneumonia and sepsis | 24h |
| *No CAA* | AFM case 1 | 65F | Lewy Body Disease, no CAA | End-stage dementia | 17h |
| *No CAA* | AFM case 2 | 27M | None | Complications of pulmonary hypertension | 11h |
| *No CAA* | AFM case 3 | 78M | Braak stage III/IV, no CAA | unknown | 20h |
| *No CAA* | AFM case 4 | 90+F | Braak stage VI, no CAA | End stage dementia | 16h |

Total of 26 cases included 11 CAA cases, 5 AD with mild CAA, 2 FTD with mild CAA, and 8 non-CAA cases without AD, or FTD. Five additional cases are included (4 non-CAA and 1 with CAA) which were included in the group assessed with atomic force microscopy but were not included primary analysis for the other figures due to the volume of available tissue. * PMI: post-mortem interval. Cerebral amyloid angiopathy (CAA); Alzheimer’s disease (AD); Atomic force microscopy (AFM).
