# Supplementary material for: Arteriolar degeneration and stiffness in cerebral amyloid angiopathy are linked to Aβ deposition and lysyl oxidase
Source: Alzheimers Dement. 2025 Jun 4;21(6):e70254. doi: 10.1002/alz.70254 (PMC12136096; doi:10.1002/alz.70254)
Supplement: Supplementary file 8 — Supporting information [file ALZ-21-e70254-s011.docx]

**Supplementary table 2 Average of vascular tracing**

| *Cases* | *CAA grades* | *Tortuosity* | *SD of Diameter* | *Avg. Diameter µm* | *Vessel length µm* |
| --- | --- | --- | --- | --- | --- |
| *Severe CAA1* | Grade 3 | 1.228 | 11.269 | 41.245 | 1184.5428 |
| *Severe CAA 2* | Grade 2 and 3 | 1.292 | 15.098 | 86.509 | 1446.7313 |
| *Severe CAA 3* | Grade 4 and 5 | 1.313 | 14.255 | 74.757 | 1353.1763 |
| *Severe CAA 4* | Grade 2 and 3 | 1.126 | 10.621 | 52.572 | 1189.6472 |
| *Severe CAA 5* | Grade 5 | 1.095 | 22.708 | 65.047 | 1094.6128 |
| *Severe CAA 6* | Grade 3 | 1.207 | 9.150 | 37.361 | 721.23543 |
| *Severe CAA 7* | Grade 2 and 3 | 1.128 | 10.615 | 41.025 | 1936.4815 |
| *Severe CAA 8* | Grade 2 and 3 | 1.337 | 30.335 | 72.428 | 1117.2443 |
| *Severe CAA 9* | Grade 3 | 1.242 | 16.422 | 49.626 | 1550.9087 |
| *Severe CAA 10* | Grade 1 and 2 | 1.119 | 13.538 | 61.611 | 1270.6133 |
| *Severe CAA 11* | Grade 1 and 2 | 1.041 | 28.627 | 129.898 | 1373.4366 |
| *CAA/AD 1* | Grade 2 | 1.129 | 8.399 | 42.125 | 1311.4108 |
| *CAA/AD 2* | Grade 1 | 1.212 | 7.687 | 37.266 | 1468.3261 |
| *CAA/AD 3* | Grade 3 | 1.340 | 10.374 | 43.079 | 1808.241 |
| *CAA/AD 4* | Grade 1 | 1.437 | 11.766 | 61.280 | 1166.5144 |
| *CAA/mixed 5* | Grade 3 | 1.199 | 7.998 | 39.992 | 1038.9729 |
| *CAA/FTD 6* | Grade 1 and 3 | 1.133 | 14.421 | 57.440 | 1543.2614 |
| *CAA/FTD 7* | Grade 1 and 3 | 1.305 | 6.921 | 33.566 | 1947.288 |
| *Control 1* | Grade 0 | 1.252 | 7.388 | 44.835 | 1564.0481 |
| *Control 2* | Grade 0 | 1.132 | 7.200 | 22.931 | 1033.110 |
| *Control 3* | Grade 0 | 1.165 | 11.127 | 35.236 | 1508.5254 |
| *Control 4* | Grade 0 | 1.052 | 5.399 | 43.644 | 944.1893 |
| *Control 5* | Grade 0 | 1.171 | 11.725 | 38.542 | 1490.8495 |
| *Control 6* | Grade 0 | 1.033 | 7.174 | 35.993 | 985.287 |
| *Control 7* | Grade 0 | 1.163 | 7.597 | 36.852 | 1512.4164 |
| *Control 8* | Grade 0 | 1.092 | 9.128 | 38.453 | 1613.9934 |

Average per case of morphological level (described in figure 2), average of diameter and length.
