# Supplementary material for: Arteriolar degeneration and stiffness in cerebral amyloid angiopathy are linked to Aβ deposition and lysyl oxidase
Source: Alzheimers Dement. 2025 Jun 4;21(6):e70254. doi: 10.1002/alz.70254 (PMC12136096; doi:10.1002/alz.70254)
Supplement: Supplementary file 9 — Supporting information [file ALZ-21-e70254-s003.docx]

**Supplementary table 3**

**Average vascular volumes of β-amyloid, lysyl oxidase, and vascular smooth muscle per patient.**

| *Category* | *Case #* | *Penetrating vessel #* | *Case#* |  | *% vascular*  *Aβ volume* | *% vascular*  *Lox volume* | *% VSMCs/Arteriole* |
| --- | --- | --- | --- | --- | --- | --- | --- |
| *Severe CAA1* | CAA1 | 19 | CAA1 |  | 15.08 ± 3.10% | 13.20 ± 4.54% | 39.7 ± 8.07% |
| *Severe CAA 2* | CAA 2 | 15 | CAA 2 |  | 42.60 ± 5.54% | 27.70 ± 5.73% | 11.02 ± 2.89% |
| *Severe CAA 3* | CAA 3 | 24 | CAA 3 |  | 13.89 ± 3.34% | 17.91 ± 4.58% | 47.59 ± 5.46% |
| *Severe CAA 4* | CAA 4 | 17 | CAA 4 |  | 10.46 ± 3.68% | 6.00 ± 3.44% | 47.41 ± 8.42% |
| *Severe CAA 5* | CAA 5 | 23 | CAA 5 |  | 26.94 ± 4.07% | 8.26 ± 2.15% | 14.52 ± 3.78% |
| *Severe CAA 6* | CAA 6 | 9 | CAA 6 |  | 1.48 ± 1.10% | 6.74 ± 5.65% | 86.62 ± 2.85% |
| *Severe CAA 7* | CAA 7 | 7 | CAA 7 |  | 65.11 ± 9.71% | 0.93 ± 0.52% | 16.95 ± 7.83% |
| *Severe CAA 8* | CAA 8 | 8 | CAA 8 |  | 17.05 ± 6.66% | 3.17 ± 3.06% | 44.75 ± 13.66% |
| *Severe CAA 9* | CAA 9 | 16 | CAA 9 |  | 13.91 ± 5.96% | 2.52 ± 1.23% | 49.72 ± 10.42% |
| *Severe CAA 10* | CAA 10 | 22 | CAA 10 |  | 64.37 ± 4.77% | 23.27 ± 6.11% | 10.52 ± 4.72% |
| *Severe CAA 11* | CAA 11 | 7 | CAA 11 |  | 23.72 ± 8.70% | 2.50 ± 1.30% | 21.21 ± 8.25% |
| *CAA/AD 1* | AD 1 | 17 | AD 1 |  | 16.97 ± 3.02 % | 13.17 ± 3.20% | 37.91 ± 5.70% |
| *CAA/AD 2* | AD 2 | 8 | AD 2 |  | 0.99 ± 0.65% | 0.23 ± 0.23% | 73.40 ± 6.76% |
| *CAA/AD 3* | AD 3 | 5 | AD 3 |  | 23.56±8.69% | 0.52±0.51% | 0.20 ± 0.14% |
| *CAA/AD 4* | AD 4 | 12 | AD 4 |  | 19.01 ± 7.12% | 14.34 ± 6.26% | 25.03 ± 5.56% |
| *CAA/mixed 5* | AD 5 | 48 | AD 5 |  | 25.74 ± 3.62% | 8.63 ± 1.99% | 22.00 ± 2.96% |
| *CAA/FTD 6* | FTD 1 | 16 | FTD 1 |  | 23.73 ± 7.91% | 9.84 ± 5.37% | 38.58 ± 8.23% |
| *CAA/FTD 7* | FTD 2 | 7 | FTD 2 |  | 21.79 ± 9.97% | 0.63 ± 0.37% | 38.44 ± 9.21% |
| *Control* | Control 1 | 8 | Control 1 |  | 3.91 ± 12.80% | 0.01 ± 0.01% | 73.67 ± 9.29% |
| *Control* | Control 2 | 15 | Control 2 |  | 0.01 ± 0.00% | 0.00 ± 0.00% | 78.47 ± 8.86% |
| *Control* | Control 3 | 22 | Control 3 |  | 13.50 ± 2.73% | 0.94 ± 0.31% | 57.53 ± 4.85% |
| *Control* | Control 4 | 10 | Control 4 |  | 1.68 ± 0.56% | 0.21 ± 07% | 79.71 ± 5.86% |
| *Control* | Control 5 | 20 | Control 5 |  | 10.01 ± 3.49% | 4.08 ± 3.34% | 44.61 ± 5.91% |
| *Control* | Control 6 | 10 | Control 6 |  | 13.14 ± 3.20% | 14.03 ± 7.27% | 4.84 ± 0.43% |
| *Control* | Control 7 | 8 | Control 7 |  | 0.13 ± 0.08% | 0.49 ± 0.15% | 64.91 ± 5.75% |
| *Control* | Control 8 | 13 | Control 8 |  | 1.06 ± 0.57% | 0.08 ± 0.02% | 102.38 ± 22.74% |

Data is presented as percent coverage (volume/vascular volume) on penetrating arterioles. The values correspond to the average per case.
