# Supplementary material for: Arteriolar degeneration and stiffness in cerebral amyloid angiopathy are linked to Aβ deposition and lysyl oxidase
Source: Alzheimers Dement. 2025 Jun 4;21(6):e70254. doi: 10.1002/alz.70254 (PMC12136096; doi:10.1002/alz.70254)
Supplement: Supplementary file 10 — Supporting information [file ALZ-21-e70254-s004.docx]

**Supplementary table 4 Spearman correlation detail for all observation**

| Log (Avg. Diameter (µm)) = 3.6127181 – 0.1553757*Log(VSM volume/Volume arterioles) Figure 2E | | | | | |  |
| --- | --- | --- | --- | --- | --- | --- |
| Cases | **n** | **Spearman correlation** | | **power fit** | **p** | **r^2^** |
| *Mild\moderate CAA donors* | 86 | -0.2387 | 0.0268 | -0.104 ± 0.049 | 0.0386 | 0.049 |
| *Severe CAA donors* | 118 | -0.4110 | <0.0001 | -0.107 ± 0.029 | 0.0004 | 0.104 |
| *All CAA cases* | 204 | -0.3706 | <0.0001 | -0.115 ± 0.024 | <0.0001 | 0.100 |
| *Neurological controls* | 76 | -0.2618 | 0.0223 | -0.250 ± 0.076 | 0.0017 | 0.125 |
| *All cases* | 281 | -0.4486 | <0.0001 | -0.155 ± 0.021 | <0.0001 | 0.159 |
|  |  |  |  |  |  |  |
| Log (Standard deviation of diameter) = 2.0211657 – 0.156574*Log(VSM volume/Volume arterioles) Figure 2E | | | | | | |
| *Mild\moderate CAA donors* | 87 | ns |  | 2.016 ± 0.101 | 0.3074 | 0.012 |
| *Severe CAA donors* | 116 | -0.2314 | 0.0117 | 2.239 ± 0.101 | 0.0095 | 0.057 |
| *All CAA cases* | 203 | -0.2232 | 0.0013 | 2.111 ± 0.071 | 0.0005 | 0.058 |
| *Neurological controls* | 78 | ns |  | 1.890 ± 0.086 | 0.0893 | 0.037 |
| *All cases* | 281 | -0.2995 | <0.0001 | 2.021 ± 0.055 | >0.0001 | 0.081 |
|  |  |  |  |  |  |  |
| Log (Tortuosity) = 0.164576 + 0.0104224*Log (VSM volume/Volume arterioles) Figure 2E | | | | |  |  |
| *Mild\moderate CAA donors* | 88 | ns |  | 0.168 ± 0.023 | 0.2279 | 0.016 |
| *Severe CAA donors* | 119 | 0.2312 | 0.0107 | 0.210 ± 0.021 | 0.0192 | 0.045 |
| *All CAA cases* | 238 | 0.1787 | 0.0096 | 0.189 ± 0.015 | 0.00178 | 0.027 |
| *Neurological controls* | 82 | ns |  | 0.129 ± 0.013 | 0.8947 | 0.000 |
| *All cases* | 289 | ns |  | 0.164 ± 0.011 | 0.1084 | 0.008 |
|  |  |  |  |  |  |  |
| Log (Vascular Aβ volume/µm3) = 10.028191 - 0.9173585*Log(VSM volume/Volume arterioles) Figure 3B | | | | | | |
| *Mild\moderate CAA donors* | 110 | -0.5264 | <0.0001 | -0.760 ± 0.158 | <0.0001 | 0.175 |
| *Severe CAA donors* | 120 | -0.6253 | <0.0001 | -0.692 ± 0.118 | <0.0001 | 0.223 |
| *All CAA cases* | 230 | -0.5961 | <0.0001 | -0.725 ± 0.092 | <0.0001 | 0.211 |
| *Neurological controls* | 69 | -0.2726 | 0.053 | -0.655 ± 0.530 | 0.2208 | 0.022 |
| *All cases* | 299 | -0.6612 | <0.0001 | -0.917 ± 0.094 | <0.0001 | 0.240 |
|  |  |  |  |  |  |  |
| Log (Vascular Aβ volume/µm3) = 4.3716951 + 1.7786531*Log(Avg. diameter (µm) Figure 3D | | | | | | |
| *Mild\moderate CAA donors* | 82 | 0.4559 | <0.0001 | 1.776 ± 0.478 | 0.0004 | 0.147 |
| *Severe CAA donors* | 113 | 0.5096 | <0.0001 | 1.372 ± 0.367 | 0.0003 | 0.111 |
| *All CAA cases* | 195 | 0.5033 | <0.0001 | 1.645 ± 0.284 | <0.0001 | 0.147 |
| *Neurological controls* | 48 | ns |  |  |  |  |
| *All cases* | 243 | 0.4869 | <0.0001 | 1.778 ± 0.286 | <0.0001 | 0.127 |
|  |  |  |  |  |  |  |
| Log (Vascular Aβ volume/µm3) = 8.8722818 + 1.0488491*Log(Standard deviation of diameter) Figure 3D | | | | | | |
| *Mild\moderate CAA donors* | 83 | 0.2300 | 0.0265 | ns |  |  |
| *Severe CAA donors* | 111 | 0.3480 | <0.0001 | 0.931 ± 0.249 | 0.0003 | 0.113 |
| *All CAA cases* | 194 | 0.3323 | <0.0001 | 0.953 ± 0.21 | <0.0001 | 0.096 |
| *Neurological controls* | 51 | ns |  |  |  |  |
| *All cases* | 245 | 0.3485 | <0.0001 | 1.048 ± 0.194 | <0.0001 | 0.106 |
|  |  |  |  |  |  |  |
| Log (Overlapped Aβ volume/µm3) = 11.17396 - 0.0818912*Log (Tortuosity) Figure 3D | | | | | |  |
| *Mild\moderate CAA donors* | *84* | *-0.2438* | *0.0268* | *ns* |  |  |
| *Severe CAA donors* | *114* | *-0.1808* | *0.0380* | *ns* |  |  |
| *All CAA cases* | *198* | *-0.1923* | *0.0038* | *ns* |  |  |
| *Neurological controls* | *54* | *ns* |  |  |  |  |
| *All cases* | *252* | *ns* |  | *ns* |  |  |
|  |  |  |  |  |  |  |
| Log (Vascular LOX volume/µm3) = 2.0222882 + 0.6568061*Log(Vascular Aβ volume/µm3) Figure 6B | | | | | | |
| *Mild\moderate CAA donors* | 94 | 0.4010 | <0.0001 | 0.427 ± 0.124 | 0.0009 | 0.113 |
| *Severe CAA donors* | 121 | 0.5690 | <0.0001 | 0.856 ± 0.126 | <0.0001 | 0.277 |
| *All CAA cases* | 215 | 0.5055 | <0.0001 | 0.635 ± 0.088 | <0.0001 | 0.193 |
| *Neurological controls* | 55 | 0.4850 | <0.0001 | 0.390 ± 0.103 | 0.0004 | 0.210 |
| *All cases* | 270 | 0.5869 | <0.0001 | 0.656 ± 0.065 | <0.0001 | 0.271 |
|  |  |  |  |  |  |  |
| Log (Vascular LOX volume/µm3) = 8.2125305 - 0.845455*Log(VSM volume/Volume arterioles) Figure 6C | | | | | | |
| *Mild\moderate CAA donors* | 94 | ns |  |  |  |  |
| *Severe CAA donors* | 117 | -0.3682 | <0.0001 | -0.653 ± 0.199 | 0.0014 | 0.085 |
| *All CAA cases* | 211 | -0.2908 | <0.0001 | -0.502 ± 0.155 | 0.0014 | 0.047 |
| *Neurological controls* | 67 | ns |  |  |  |  |
| *All cases* | 278 | -0.4142 | <0.0001 | -0.845 ± 0.135 | <0.0001 | 0.124 |
|  |  |  |  |  |  |  |
| Log (Vascular LOX volume/µm3) = 3.7015854 + 1.5044236*Log(Avg. diameter (µm) Figure 6D | | | | | | |
| *Mild\moderate CAA donors* | 233 | ns |  |  |  |  |
| *Severe CAA donors* | 111 | 0.2375 | 0.0065 | ns |  |  |
| *All CAA cases* | 183 | 0.2214 | 0.0009 | ns |  |  |
| *Neurological controls* | 50 | ns |  |  |  |  |
| *All cases* | 233 | 0.2856 | <0.0001 |  |  |  |
|  |  |  |  |  |  |  |
| Log (Vascular LOX volume/µm3) = 6.8729659 + 1.1202172*Log(Standard deviation of diameter) Figure 6D | | | | | | |
| *Mild\moderate CAA donors* | 235 | ns |  |  |  |  |
| *Severe CAA donors* | 109 | 0.3325 | 0.0001 | 1.158 ± 0.391 | 0.0038 | 0.075 |
| *All CAA cases* | 182 | 0.2542 | 0.0001 | 1.002 ± 0.300 | 0.0001 | 0.058 |
| *Neurological controls* | 53 | ns |  |  |  |  |
| *All cases* | 235 | 0.2759 | <0.0001 | 1.120 ± 0.258 | <0.0001 | 0.074 |
|  |  |  |  |  |  |  |
| Log (Vascular LOX volume/µm3) = 9.5300615 - 1.1765328*Log (Tortuosity index) Figure 6D | | | | | |  |
| *Mild\moderate CAA donors* | 74 | -0.2287 | 0.0274 | ns |  |  |
| *Severe CAA donors* | 112 | ns |  |  |  |  |
| *All CAA cases* | 186 | ns |  |  |  |  |
| *Neurological controls* | 56 | ns |  |  |  |  |
| *All cases* | 242 | ns |  |  |  |  |

Correlation for detail for all observation for the corresponding figures in detail.
